# Scientific contributions of citizen science applied to rare or threatened animals

**DOI:** 10.1111/cobi.13976

**Published:** 2022-10-13

**Authors:** Amélie Fontaine, Anouk Simard, Nicolas Brunet, Kyle H. Elliott

**Affiliations:** ^1^ Department of Natural Resource Sciences McGill University Sainte‐Anne‐de‐Bellevue Quebec Canada; ^2^ Ministère de la forêt, de la faune et des parcs Québec Quebec Canada; ^3^ School of Environmental Design and Rural Development University of Guelph Guelph Ontario Canada

**Keywords:** collaboration type, conservation measure, data quality, gray literature, longevity, participatory science, scientific publication, calidad de datos, ciencia participativa, literatura gris, longevidad, medida de conservación, publicación científica, tipo de colaboración, 保护措施, 数据质量, 灰色文献, 持续时间, 参与式科学, 科学出版物

## Abstract

Citizen science is filling important monitoring gaps and thus contributing to the conservation of rare or threatened animals. However, most researchers have used peer‐reviewed publications to evaluate citizen science contributions. We quantified a larger spectrum of citizen science's contributions to the monitoring of rare or threatened animals, including contributions to the peer‐reviewed publications, gray literature and to conservation measures (i.e., actions taken as a direct result of citizen science monitoring). We sought to provide broad information on how results of studies of citizen science monitoring is used. We also evaluated factors associated with success of citizen science projects. We conducted a web search to find citizen science projects focusing on rare and threatened species and surveyed citizen science project managers about their contributions and factors influencing their success. The number of projects increased rapidly after 2010. Almost one‐half of the citizen science projects produced at least 1 peer‐reviewed publication, 64% produced at least 1 gray literature publication, and 64% resulted in at least 1 conservation measure. Conservation measures covered a wide range of actions, including management and mitigation plans, modification of threat status, identification and establishment of protected areas, habitat restoration, control of invasive species, captive breeding programs, and awareness campaigns. Longevity, data quality, and collaboration type best explained quantities of all types of scientific contributions of citizen science. We found that citizen science contributed substantially to knowledge advancement and conservation, especially when programs were long term and had rigorous data collection and management standards, and multidisciplinary or transdisciplinary collaborations.

## Resumen

La ciencia ciudadana contribuye a llenar vacíos en el monitoreo, lo que ayuda a la conservación de animales raros o amenazados. Sin embargo, la mayoría de los investigadores han usado publicaciones revisadas por pares para evaluar las contribuciones de la ciencia ciudadana. Cuantificamos un mayor espectro de las contribuciones de la ciencia ciudadana al monitoreo de animales raros y amenazados, incluyendo las contribuciones a la literatura gris, a las publicaciones revisada por pares y a las medidas de conservación (es decir, las acciones tomadas como resultado directo del monitoreo ciudadano). Buscamos proporcionar información generalizada sobre cómo los resultados de los estudios de monitoreo ciudadano es usado. También evaluamos los factores asociados con el éxito de los proyectos de ciencia ciudadana. Realizamos una búsqueda en línea para encontrar proyectos de ciencia ciudadana enfocados en especies raras o amenazadas y encuestamos a los gestores de estos proyectos sobre sus contribuciones y los factores que influyen sobre su éxito. El número de proyectos incrementó rápidamente a partir de 2010. De los proyectos de ciencia ciudadana, casi la mitad produjo al menos 1 publicación revisada por pares, el 64% produjo al menos una publicación en la literatura gris y el 64% derivó en al menos 1 medida de conservación. Las medidas de conservación abarcaron una gama extensa de acciones que incluyeron planes de gestión y mitigación, modificación del estado de amenaza, identificación y establecimiento de áreas protegidas, restauración del hábitat, control de especies invasoras, programas de reproducción en cautiverio y campañas de concientización. La longevidad, calidad de los datos y el tipo de colaboración explicaron de mejor manera las cantidades de todos los tipos de contribuciones científicas hechas por la ciencia ciudadana. Descubrimos que la ciencia ciudadana contribuyó sustancialmente al avance del conocimiento y la conservación, especialmente cuando los programas eran a largo plazo y contaban con estándares rigurosos de recolección y gestión de datos, y con colaboraciones multi o transdisciplinarias.

## 【摘要】

公民科学正在填补监测上的重大空缺, 从而为珍稀或受威胁动物的保护做出贡献。然而, 大多数研究者仅用同行评议文献来评估公民科学的贡献。我们量化了公民科学在监测珍稀或受威胁动物中更大范围的贡献, 包括对同行评议的灰色文献和保护措施(即作为公民科学监测的直接结果而采取的行动)的贡献, 从而为优化使用公民科学监测研究结果提供更多信息。我们还评估了影响公民科学项目成功的因素。本研究通过网络搜索寻找关注珍稀和受威胁物种的公民科学项目, 并访问了项目管理者, 以了解这些项目的贡献以及影响其成功的因素。我们发现, 公民科学项目的数量在2010年后迅速增加。几乎有一半的公民科学项目发表了至少一篇同行评议的文章, 64%的项目发表了至少一篇灰色文献, 还有64%的项目制定了至少一项保护措施。保护措施涵盖的行动十分广泛, 包括管理和减缓影响计划、改变威胁状态、识别和建立保护区、恢复生境、控制入侵物种、圈养繁殖计划和宣传活动。持续时间、数据质量和合作类型能最好地解释公民科学的各类科学贡献的数量。结果表明, 公民科学对知识进步和保护做出了巨大贡献, 特别是长期、有严格的数据收集与管理标准, 以及多学科或跨学科合作的项目。**【翻译:胡怡思;审校:聂永刚】**

## INTRODUCTION

Long‐term and large‐scale monitoring of wildlife populations is fundamental to learn more about subjects relevant to conservation, such as species range shifts, phenology, changes in community composition, and ecology of infectious disease (e.g., Dickinson et al., 2010; Nielsen et al., 2009; Sinclair et al., 2006). Such monitoring is crucial for understanding drivers of decline of rare or threatened species and for incorporating population trends into policy frameworks for legislative protection (Robinson et al., 2018). However, monitoring is difficult for such species because data are usually sparse and require more effort to collect than for common species. Traditional sampling approaches have limited value for rare and threatened species because a very low proportion of randomly chosen sampling sites, even in the appropriate habitat, are likely to be inhabited by those species (Green & Young, 1993; Rushton et al., 2004). Alternative sampling plans exist for rare or elusive species, but abundance‐estimation methods are challenging (Specht et al., 2017; Thompson, 2013).

Citizen science is defined as production of scientific knowledge by actors from civil society (individuals or groups without regard to official citizenship; Houllier & Merilhou‐Goudard, 2016). Citizen science contributes to a diversity of outcomes and is particularly valuable for collecting data or monitoring trends in rare or threatened species. Citizen science projects can focus on a particular area where the rare or threatened species is located, employing systematic monitoring and complex approaches, or can occur across a large extent, employing mass participation and simple approaches (Pocock et al., 2017). Examples include monitoring monarch butterfly (*Danaus plexippus*) migration (monarchjointventure.org), the arrival of white‐nose syndrome in bats (batwatch.ca), or tracking movements of humpback whales (*Megaptera novaeangliae*) (caribtails.org).

Several studies highlight the educational value of citizen science (Bell et al., 2009; Bonney et al., 2009; Dickinson et al., 2012; Feldman et al., 2021; Follet & Strezov, 2015; Pocock et al., 2019). Indeed, citizen science helps democratize science, builds social capital, engages the general public in planning and management of local ecosystems, and provides opportunities for participants to gain scientific knowledge, connect with nature, and develop positive attitudes toward science (Bonney et al., 2009; Miller, 2005; Pollock & Whitelaw, 2005; Schwartz, 2006). Despite past skepticism, mainly related to data reliability (Cohn, 2008; Dickinson et al., 2010), citizen science projects that address a variety of ecological and environmental questions are increasing worldwide (Conrad & Hilchey, 2011; Pocock et al., 2017) and are providing increasing evidence that citizen science can produce high‐quality data and be educational for participants (Kosmala et al., 2016; McKinley et al., 2017).

Citizen science projects can generate large data sets, often with wide monitoring coverage at low cost, that allow scientists and members of the public to consider ecological questions that would be otherwise impossible to answer (Conrad & Hilchey, 2011; Tulloch et al., 2013). However, obtaining reliable scientific results from citizen science data may require new analytical tools designed for big data sets that can address problems of data fragmentation or inaccuracy, monitoring biases, lack of experimental design, or insufficient quality control (Conrad & Hilchey, 2011).

Few studies have assessed the impacts of citizen science on ecological research, and those studies that have are limited in scope because they primarily examined peer‐reviewed publications (Chandler, See, et al., 2017; Conrad & Hilchey, 2011; Dickinson et al., 2012). Thus, other types of scientific contributions, such as gray literature publications (government reports, conference proceedings, conference abstracts, book chapters, theses, and magazine articles) and actions taken to minimize direct and indirect negative impacts on threatened and species and their habitats, such as management plans and protection of breeding grounds (hereafter conservation measures), are largely absent in the current literature. Yet, these contributions are critical for the conservation of rare or threatened species. Indeed, when actions are urgently needed to manage a species, publishing required information in the peer‐reviewed literature may take too long. Raw data or gray literature publications may be more readily accessible and can thus be used to take quick action (Castellanos‐Galindo et al., 2011; Guisan et al., 2013; Sullivan et al., 2006). Peer‐reviewed publications, the gray literature, and conservation measures are all important types of scientific contributions in monitoring and conservation of rare or threatened species. To date, there has been no global quantification of citizen science contributions to scientific knowledge and its impact on conservation that has included an examination of peer‐reviewed publications, the gray literature, and conservation measures combined. Although there are many examples of data collected by citizen science projects that have played a crucial role in answering basic and applied ecological questions (Barlow et al., 2015; Dickinson et al., 2012; Gardner et al., 2019), it remains unclear what differentiates successful from unsuccessful projects in terms of the number of scientific contributions (i.e., peer‐reviewed publications, gray literature publications, and conservation measures). For example, the focal taxon may affect the number of conservation measures undertaken in a project. Projects focused on charismatic species may produce more conservation measures. Project longevity may also affect all types of scientific contributions, with contributions increasing through time.

Because the quantification of citizen science contributions to wildlife conservation in peer‐reviewed publications is scarce and its quantification in the gray literature and quantification of conservation measures are largely absent in the peer‐reviewed literature, we assessed these contributions to the conservation of rare and threatened animals based on all 3 sources to provide a more complete picture of citizen science conservation contributions. Although opportunistic multispecies monitoring schemes (e.g., eBird, iNaturalist, Christmas Bird Counts, bioblitzes) make diverse scientific contributions, such as expanding scientific knowledge and scientific literacy (Bonney et al., 2009), we were interested in the growing multitude of citizen science projects that focus on the monitoring and conservation of specific rare or threatened animals. We addressed 3 questions: What are the spatial and temporal trends of citizen science projects that focus on rare or threatened animals? What are the scientific contributions to wildlife conservation these projects produce based on the number of peer‐reviewed publications, gray literature publications, and direct contributions to conservation (i.e., conservation measures)? What variables predict the success of those projects in terms of the number of scientific contributions? We expected the cumulative number of projects to increase through time and citizen science projects to contribute more to the gray literature and conservation measures than to peer‐reviewed publications. We hypothesized that a range of factors (e.g., collaboration type and data quality) predict success of citizen science contributions (full list of predictions in Appendix S5).

## METHODS

### Search for citizen science projects

To explore the spatial and temporal trends of citizen science applied to rare or threatened animals and assess the scientific contributions of such projects, we searched online for citizen science projects and created a global database of such projects. We used search terms such as *citizen science* OR particip* and *rare* OR *threatened*. We selected terms based on studies in which a similar method was used (e.g., Pocock et al., 2017; Theobald et al., 2015) and our knowledge of relevant keywords. Search terms such as *bat* OR chiropter* were included in the English search (Appendix S1). Using the Google search engine, we selected the options “pages in English language only” and “personalized dates from 2019 and before.” To expand our search, as advised by Nuñez and Amano (2021), we applied the same method to search Spanish and French language pages. We used the same method and the Baidu search engine to locate Chinese projects. While acknowledging that the success of many projects may rely on communication in local languages and dialects, we believe that choosing the languages spoken by the most people worldwide provided global representation. A full list of search terms in English, Spanish, French, and Chinese is in Appendix S1.

We opened the first 100 links produced by the search, which included but were not restricted to major citizen science directories, such as scistarter.com, zooniverse.org, and CitSci.org. We used a snowball sampling method to discover more links. We included in our database projects that matched the following 4 criteria: project managers requested public assistance to monitor animals or otherwise further the scientific process (e.g., sorting images); focal animals were locally or globally considered as rare, threatened, or endangered (as usually mentioned on the website and confirmed via an external source, such as the International Union for Conservation of Nature Red List, national or local lists; if the program had many focal species, over one‐half the species studied needed to be rare or threatened for inclusion); the project's aim included the monitoring or conservation of those animals (e.g., projects that were exclusively about education and communication were excluded); and project ran for ≥2 years. We screened over 5600 links in 4 different languages. A total of 332 projects fit our criteria. For each of these, we identified the goal and type of data collected of each project.

### Survey of project managers to assess scientific contributions and predictors of success

For each project in our database, we assessed the number of articles published in peer‐review journals; number of publications resulting from the project in the gray literature; number of conservation measures that resulted directly from the project; and predictors of success (e.g., project location, focal taxa, collaboration type) through a survey of managers or coordinators of citizen science projects with a website in English. Survey questions are in Appendix S17.

In the survey, we provided a definition for each scientific contribution to remove ambiguity. We did not, however, define *peer‐reviewed journal publication* because the meaning is known to survey participants. Publications in the gray literature were defined as government reports, conference proceedings, conference abstracts, book chapters, theses, and magazine articles. Conservation measures were defined as actions taken that minimized direct and indirect negative impacts on a species of conservation concern and its habitat with the goal of sustaining the natural value of an area and the existence of a species, respectively (Sale, 2002). Direct contributions to conservation included official policies or publications, consisting of conservation initiatives, management decisions, or policy actions, such as the assessment of the local or global conservation status of a species or land‐use restrictions. To facilitate the survey, we classified scientific contributions into 5 categories: none, 1, 2−5, 6−10, or >10 scientific contributions. To corroborate and supplement our results, we also visited the websites and searched for additional information on all projects in the candidate list.

### Assessment of predictors of success

For each surveyed project, we assessed the following variables that could predict its success in terms of the 3 scientific contributions mentioned above: project longevity (time since the project foundation [range 1945−2019]); location (North America, Central America, South America, Africa, Europe, Russia, Asia, Oceania, Arctic or Antarctica, worldwide); scale of data collection (local, regional, national, international); focal taxa (mammal, bird, reptile or amphibian, fish, invertebrate, mixed of more >1 taxon); collaboration type (intra‐, multi‐, inter‐, or transdisciplinary); organization type (citizen driven, nonprofit, academic, governmental, industrial, mixed of 2, mix of 3 or more); methodological complexity (used to evaluate factors affecting data quality only and assessed as in Pocock et al. [2017]); and data quality (predictor of success [details in Appendix S2]). We collated survey answers and website content and only included projects for which we had all the information needed for analyses of predictors of success.

Collaboration types were defined as follows: intradisciplinary, working within a single discipline (e.g., biology); multidisciplinary, people from different disciplines working together, each drawing on their disciplinary knowledge in an additive manner (e.g., studying bats from the morphology and disease‐carrying perspective); interdisciplinary, integration of knowledge and methods from different disciplines through the use of a synthesis of approaches with a strong level of cooperation; and transdisciplinary, creation of a unity of intellectual frameworks beyond the disciplinary perspectives to form a new holistic approach.

Data quality was assessed by attributing a score based on the criteria in Theobald et al. (2015) and Kosmala et al. (2016). These criteria increase data quality by boosting data accuracy and accounting for bias. We selected 7 criteria that were the most important and common to the 2 studies and that would be easy to understand by our survey respondents. The criteria were as follows: protocol freely available, support and training provided, data entry type, recording of metadata, data verifiability, and data validation and standardization (Appendix S3). Each criterion was scored on a scale of 0−1.5 or as not applicable. Maximum possible score was 10.5. We normalized this score to 1 to create a data quality index that ranged from 0 to 1 for each project.

This study was conducted under a certificate of ethical acceptability of research involving humans (2003038) and adhered to guidelines from the McGill University research ethics board.

### Statistical analyses

We assessed whether trends in the number of projects per year varied by location with a general linear model with a first‐degree polynomial function and a quasi‐Poisson distribution. We followed this with a Tukey test (Appendix S4). We used the Pearson correlation coefficient (with a value from –1 to 1) to measure the linear association among scientific contribution types. We tested the influence of predictors on each scientific contribution success with ordinal logistic regression models (polr from the MASS package [Venables & Ripley, 2002]). We then used a global model that included the most plausible predictors of success (Appendix S5) and used the dredge function in the MuMIn package (Barton, 2019) to generate a model selection table from the selected subset of models combining terms of the global model. We evaluated the candidate models with Akaike's information criterion corrected for small sample size (AICc) and selected the simplest model with ∆AICc < 2.00 as the best model. We interpreted predictors as having a significant statistical effect if confidence intervals excluded 0. We also used linear models (lm) to test the influence of predictors on data quality with the model selection process mentioned above (results in Appendices S12–S16). To reduce the skew in the data, we used a log_10_ transformation on the longevity and a square‐root transformation on the data quality index. We used packages listed above to carry out all analyses with R 3.5.1.

## RESULTS

**FIGURE 1:**
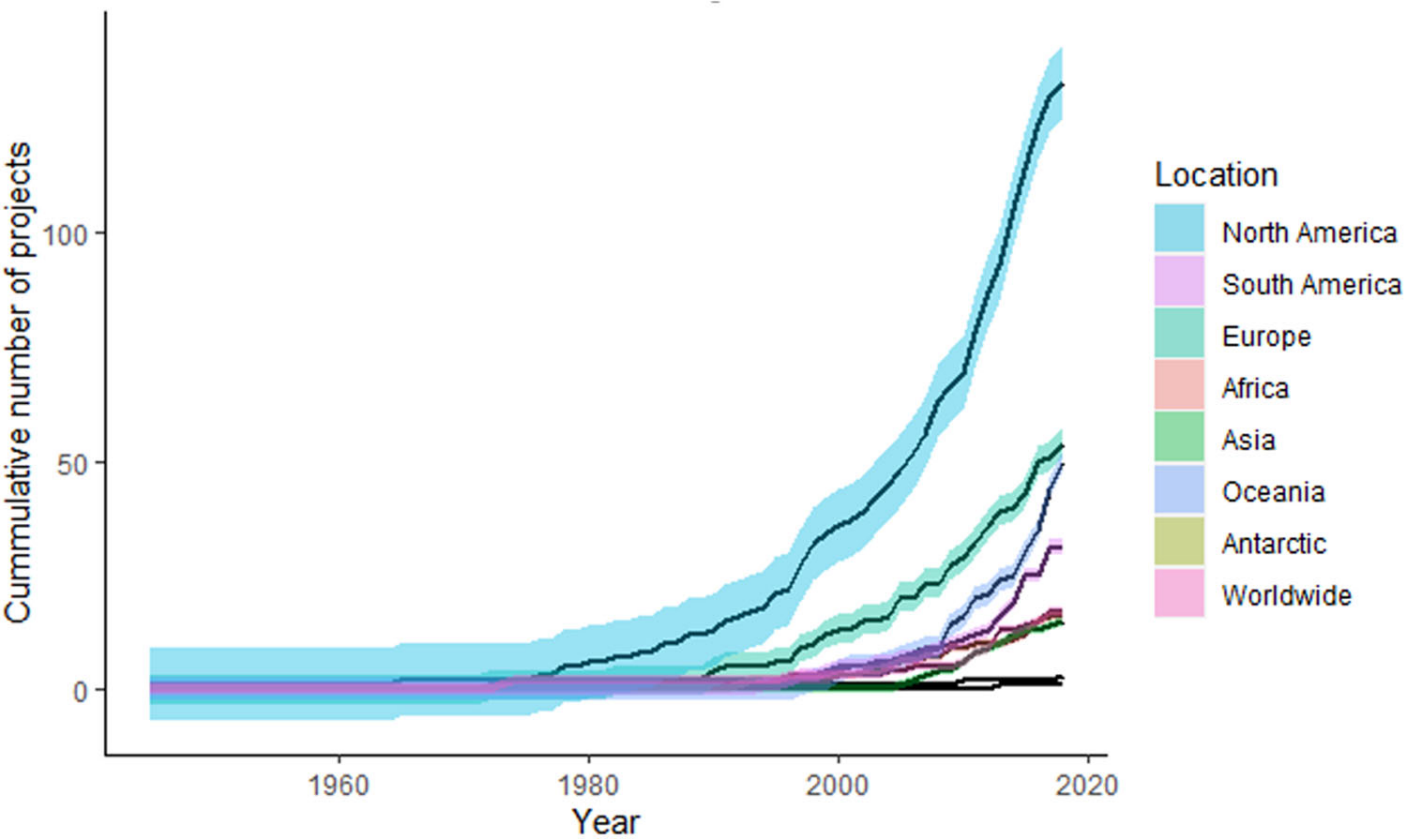
Cumulative number of citizen science projects on rare or threatened species from 1940 to 2020 (*n* = 332) (shaded areas, confidence intervals; worldwide, projects not primarily restricted to 1 continent)

**FIGURE 2:**
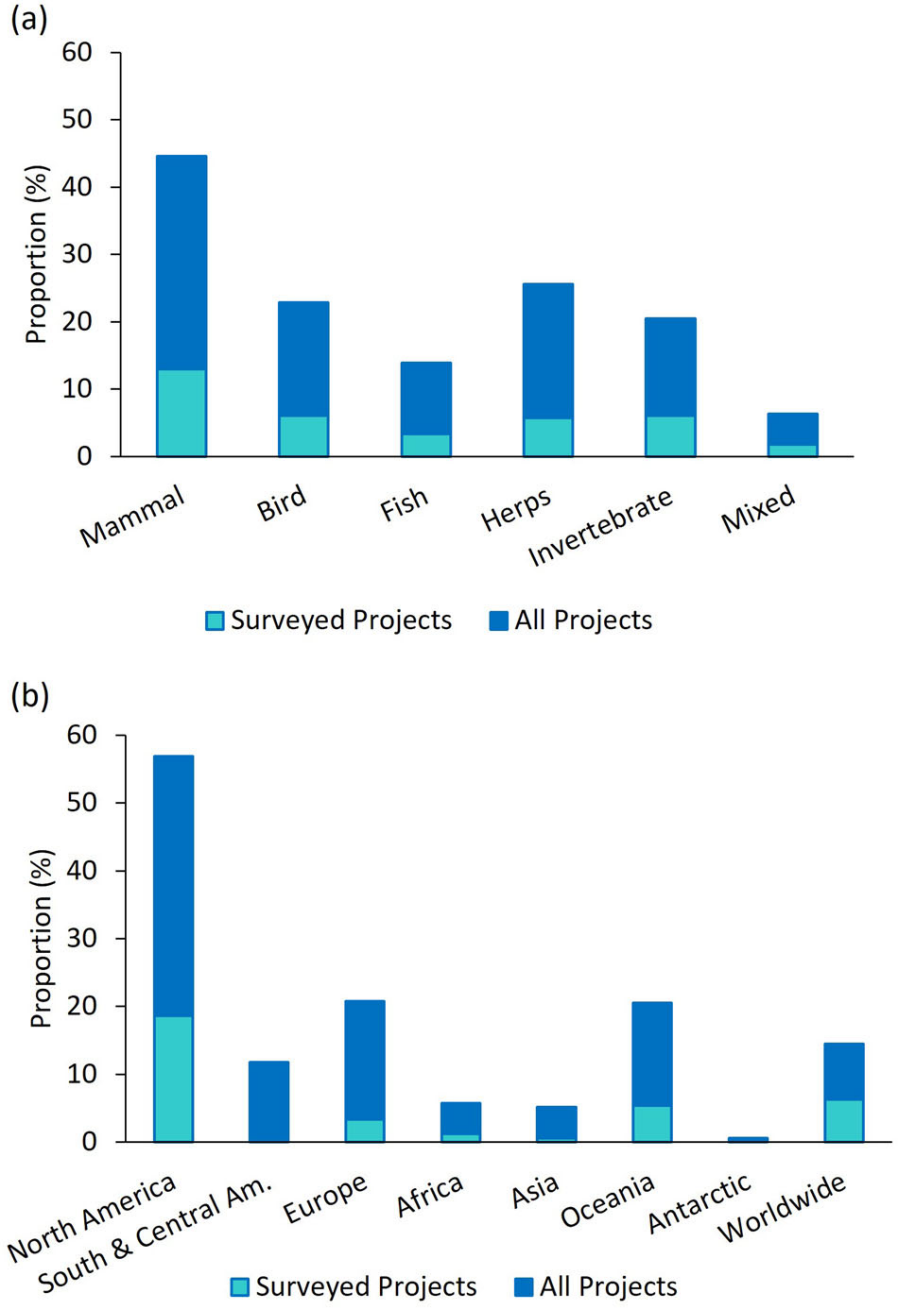
Percentage of (a) citizen science projects on rare or threatened species found online (*n* = 332) and surveyed (*n* = 119) by taxa and (b) projects on rare or threatened species found online (*n* = 332) and surveyed (*n* = 119) by location

The number of projects discovered via our internet search (90% still active in 2020) increased exponentially for most locations from 2000 to 2020 (Figure 1). Projects started earlier and grew faster in North America than other locations; a global acceleration started in 2010. In 2010, computer‐based image classification projects started. This type of project constituted 10% of those started in 2010 and after. From 235 projects with websites in English, we received 119 surveys from project managers (response rate of 51%). Projects were focused primarily on mammals, followed by reptiles and amphibians, birds, invertebrates, fishes, and a mix of species (Figure 2a). Projects were distributed across all continents; over one‐half occurred in North America (Figure 2b).

### Scientific contributions and predictors of success

**FIGURE 3:**
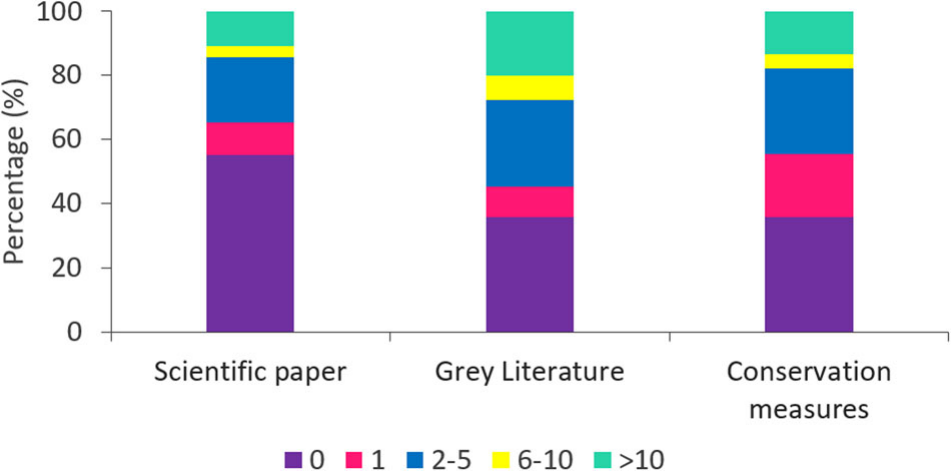
Percentage of scientific contributions of citizen science projects applied rare or threatened species surveyed (*n* = 119) by range of number of contributions (colors) for each type of contribution (conservation measures are actions taken to minimize direct and indirect negative impacts on a species of conservation concern and its habitat)

**FIGURE 4:**
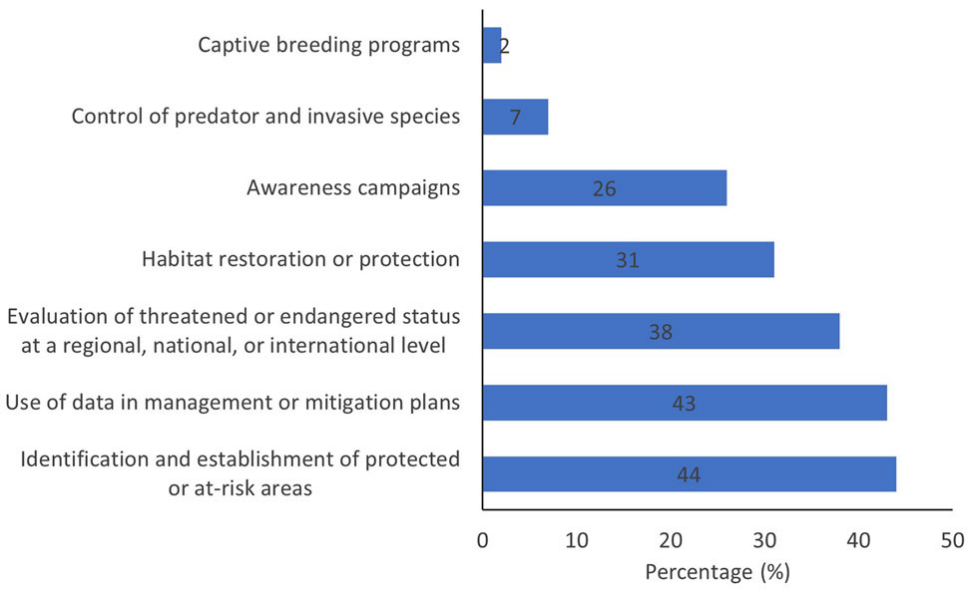
Percentage of citizen science projects on rare or threatened species resulting in conservation measures (*y*‐axis) and known to contribute to the different types of measures (*n* = 55) (numbers in bars, number of projects that have produced this type of conservation measure. More than 1 type of measure can be attributed to a project.

Surveyed projects mostly generated knowledge on population trends and species distributions, but participants also collected data on habitat selection, population dynamics, behavior, effect of threats or conservation measures on the species, and more. Generally, projects aimed for knowledge advancement, conservation, and public education. Forty‐five percent of the surveyed projects produced at least 1 peer‐reviewed scientific publication, 64% produced at least 1 publication in the gray literature, and 64% produced at least 1 conservation measure (Figure 3). Conservation measures were diverse (Figure 4). Several projects provided data to other organizations that then wrote management and mitigation plans or improved policies. Data on some species were used to evaluate threatened or endangered status at a regional, national, or international level. Data were also used to identify at‐risk areas (e.g., road mortality hotspots) and establish protected areas (e.g., protected breeding grounds). Other projects contributed directly to conservation measures, such as habitat restoration or protection, control of invasive species, captive breeding programs, and awareness campaigns. The Pearson correlation coefficient among scientific contribution types varied from 0.40 to 0.51. There was, for example, a medium correlation between the number of scientific papers and the number of conservation measures.

**FIGURE 5:**
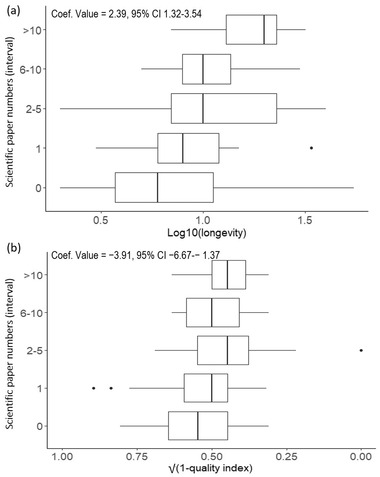
Number of peer‐reviewed scientific papers relative to (a) citizen science project longevity and (b) project data quality index (*n* = 119) (bars, interquartile range; whiskers, minimum and maximum; vertical lines in bars, lower quartile, median, and upper quartile; dots, outliers). Data transformed to achieve normality. Coefficient and *p* value derived from the ordinal logistic regression best model. Low scores on the √(1 – quality index) axis represent high data quality, and high scores represent low data quality, as assessed based on our data quality index.

**FIGURE 6:**
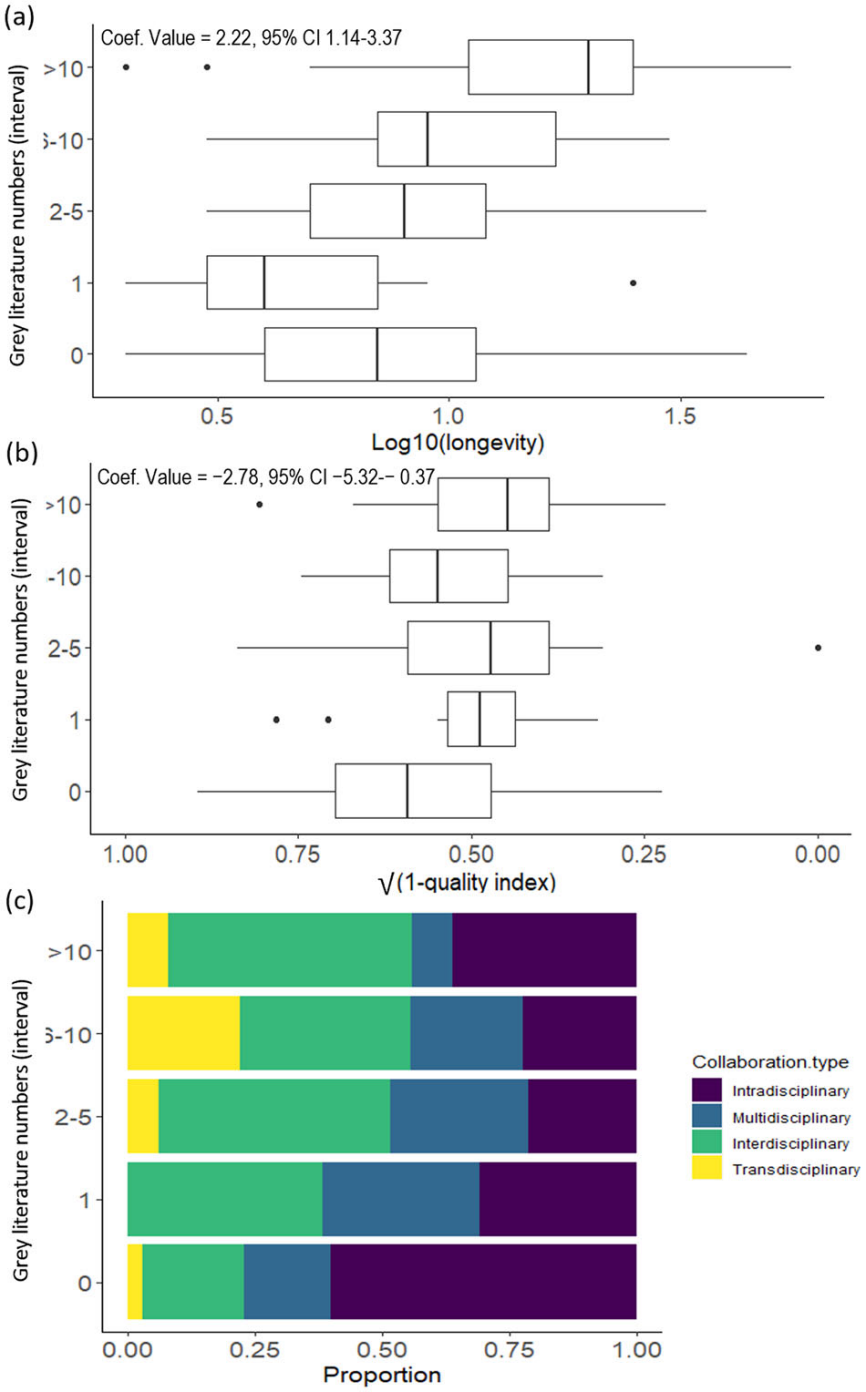
Number of gray literature publications relative to (a) project longevity, (b) project data quality index (bars, interquartile range; whiskers, minimum and maximum; vertical lines in bars, lower quartile, median, and upper quartile; dots, outliers), and (c) project collaboration type (*n* = 119). Data transformed to achieve normality. Coefficient and *p* value derived from the ordinal logistic regression best model. Low scores on the √(1 – data quality index) axis represent high data quality and high scores represent low data quality, as assessed based on our data quality index.

**FIGURE 7:**
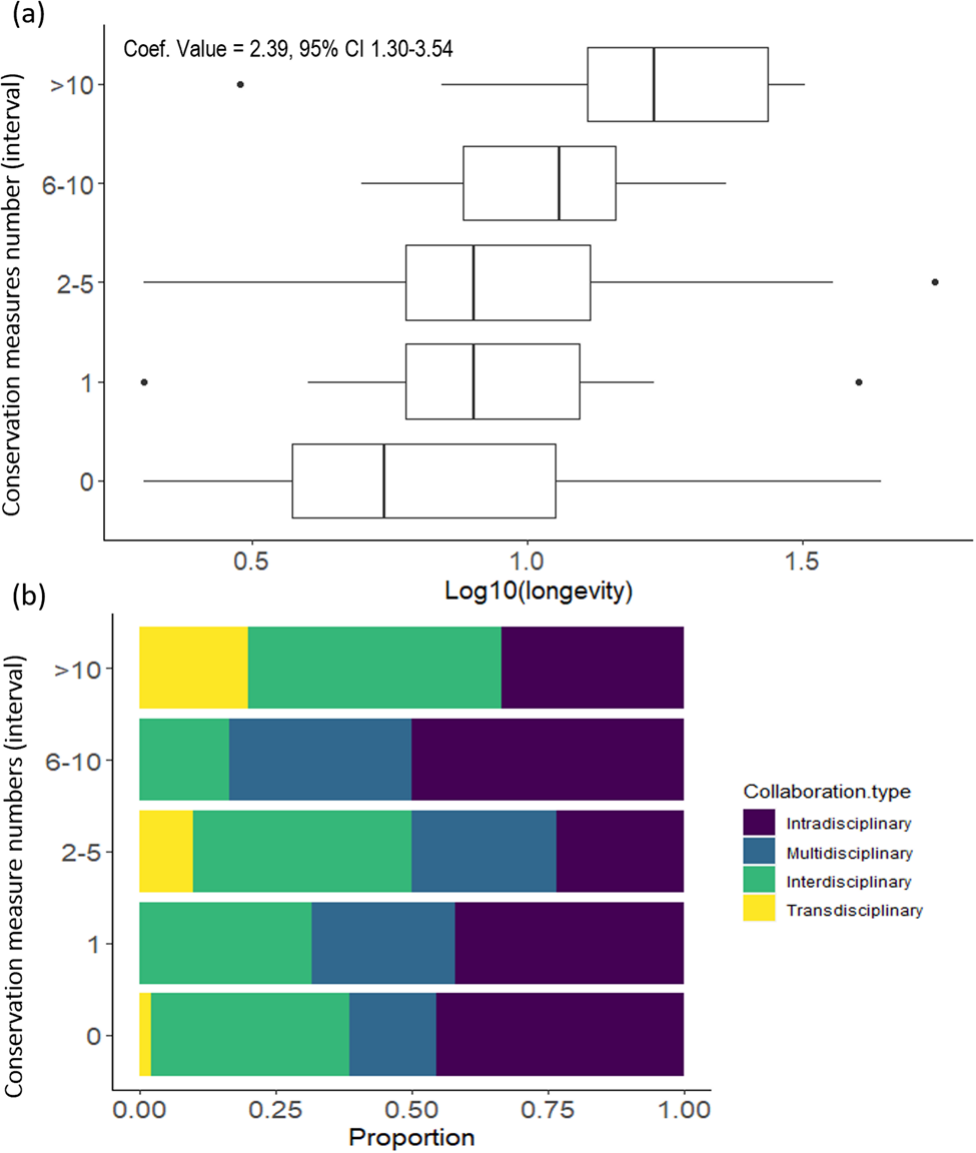
Number of conservation measures (i.e., actions taken to minimize direct and indirect negative impacts on a species of conservation concern and its habitat) relative to citizen science project (a) longevity (bars, interquartile range; whiskers, minimum and maximum; vertical lines in bars, lower quartile, median, and upper quartile; dots, outliers) and (b) collaboration type (*n* = 119). Data transformed to achieve normality. Coefficient and *p* value derived from the ordinal logistic regression best model.

The model best explaining the number of peer‐reviewed publications had a weight of 0.71, an *R* ^2^ of 0.43, and included 2 parameters: longevity and data quality index (Table 1; full list of model selection and predictor outputs is in Appendices S6 & S7). The number of scientific papers produced increased as project longevity increased (coef. value = 2.39 [95% confidence interval: 1.32–3.54]) and data quality index (coef. value = −3.91 [−6.67 to 1.37]) (Figure 5). The model best explaining the number of gray literature publications had a weight of 0.58, an *R* ^2^ of 0.35, and included 3 parameters: longevity, data quality index, and collaboration type (Table 1; full list of model selection and predictor outputs is in Appendices S8 & S9). The number of gray literature publications increased as project longevity (coef. value = 2.22 [1.14–3.37]), data quality index (coef. value = −2.78 [−5.32 to 0.37]), and projects with trans‐ and interdisciplinary collaborations increased (Figure 6). Four models explaining the number of conservation measures had ΔAICc < 2. These models all included a combination of the same 4 factors (longevity, data quality index, collaboration type, and taxa) and a cumulative weight of 0.55. Of those models, the simplest had a weight of 0.09, a *R* ^2^ of 0.66, and included 2 parameters: longevity and the collaboration type (Table 1; full list of model selection and predictor outputs is in Appendices S10 & S11). The number of conservation measures increased as project longevity (coef. value = 2.39 [1.30–3.54]) and transdisciplinarity increased (Figure 7).

**TABLE 1:** Top 5 model‐selected predictors of success of scientific contributions (peer‐reviewed publications; gray literature publications; and conservation measures^a^) of citizen science projects on rare or threatened animals ranked by their corresponding Akaike's information criterion corrected for small sample size (AICc)

Model	AICc	∆AICc^b^	*w_i_* ^c^
Peer‐reviewed publications			
log(longevity) + √(1 − quality index)	287.5	0.00	0.689
log(longevity) + √(1 − quality index) + location	290.8	3.26	0.135
log(longevity) + √(1 − quality index) + collaboration	291.5	3.98	0.094
log(longevity) + √(1 − quality index) + organisation	294.4	6.91	0.022
log(longevity)	294.7	7.19	0.019
Gray literature publications			
log(longevity) + √(1 − quality index) + collaboration	331.7	0.00	0.584
log(longevity) + √(1 − quality index)	333.9	2.15	0.199
log(longevity) + collaboration	334.5	2.76	0.147
log(longevity)	337.1	5.41	0.039
log(longevity) + √(1 − quality index) + collaboration + taxa	340.0	8.28	0.009
Conservation measures			
log(longevity) + √(1 − quality index) + collaboration + taxa	318.2	0.00	0.183
log(longevity) + collaboration + taxa	318.8	0.52	0.141
log(longevity) + √(1 − quality index) + taxa	319.0	0.78	0.124
log(longevity) + collaboration	319.8	1.52	0.085
log(longevity) + √(1 − quality index) + collaboration	319.9	1.69	0.079

^a^ Actions taken to minimize direct and indirect negative impacts on species of concern and their habitats, such as management plans and protection of breeding grounds.

^b^ Difference between AICc for the current model and the minimum of AICc among all the models.

^c^ Akaike weights.

## DISCUSSION

### Spatial and temporal trends

The number of citizen science projects studying rare or threatened species increased substantially after 2010, independent of the continent, mirroring a rise in the number of general citizen science projects (Follett & Strezov, 2015; Kullenberg & Kasperowski, 2016; Theobald et al., 2015). Pocock et al. (2017) list 3 drivers for the growth of ecological and environmental citizen science that could also be applied to our set of projects: new technologies, change in societal and cultural acceptability of different types of projects, and advances in statistical approaches. For threatened species in particular, the widespread awareness of the global loss of species and the exacerbation of this loss by increasing human impacts (Manfredo, 2008; Millard et al., 2021) may also have contributed to the rise in the number of citizen science projects.

Of the 332 projects we identified, most projects studied mammals, reptiles and amphibians, birds, and invertebrates. Presumably charismatic and easy to access and identify animals were overrepresented. Over one‐half of projects were in North America, followed by Europe and Oceania. Similarly, Cunha et al. (2017) found that 58% of publications resulting from environmental citizen science projects originated in North America. Several reasons could explain this result, such as the greater land area of North America compared with Oceania and Europe or our focus on rare or threatened wildlife may have resulted in the exclusion of projects focusing on a whole taxon, biodiversity, or the environment in general. Because there is a negative spatial relationship between the number of peer‐reviewed studies that show conservation action tests and the number of threatened and data‐deficient species (Christie et al., 2021), there is a great potential to expand citizen science outside North America, Oceania, and Europe, where most rare and threatened animals are located (Pocock et al., 2019).

### Quantification of scientific contributions

Citizen science contributes substantially to many domains of science, including wildlife conservation, natural resources, and environmental science (McKinley et al., 2017). For example, data collected on bird populations by the citizen science projects of the Cornell Lab of Ornithology have been used to examine changes in bird distribution over time and space, influences of environmental changes on breeding success, spread of emerging infectious diseases through wild animal populations, and effects of acid rain (Altizer et al., 2004; Hames et al., 2002; Rosenberg et al., 1999; Wells et al., 1998). However, few studies have quantified the global contribution of citizen science as a whole (i.e., not just contributions to the peer‐reviewed literature) and most are limited to a specific project or location (e.g., Arthur et al., 2014; Gardner et al., 2019; Poisson et al., 2020). In a survey across several groups of citizen science projects, Newman et al. (2017) found the contribution to management plans was 14% for CitSci.org projects and 50% for The Stewardship Network New England projects. The contributory and place‐based model of citizen science used by Earthwatch leads to over 60% of projects annually producing scientific publications as well as input to management plans or policies (Chandler, See, et al., 2017).

Our study allows to quantify and compare 3 types of the scientific contributions of citizen science focusing on rare or threatened species. Our Pearson correlation coefficient among scientific contribution types of 0.40–0.51 means that projects that are proficient in a specific scientific contribution type will usually be proficient, to some extent, in the other scientific contribution types, although some trade‐offs are likely to occur. As we predicted, projects contributed more to the gray literature and conservation measures than to the peer‐reviewed literature. The monitoring of threatened species often relies on governmental or nonprofit organizations whose outputs typically include reports or proposed conservation measures. Legal protection for threatened or rare species often relies on the best available science, and the contribution of raw data, gray literature, and direct mitigation actions provided by citizen science projects to species conservation should not be underestimated (Hemmi & Graham, 2014; Sullivan et al., 2006). We revealed diverse and numerous conservation measures arising from citizen science projects focusing on rare or threatened species. As shown in other studies on a specific taxon, citizen science projects focusing on rare or threatened species collect data that can be used to conserve these species or their habitats and provide for evidence‐based government policy (Greenwood, 2007; Kelly et al., 2020).

Almost one‐half of the projects included in our survey produced at least 1 peer‐reviewed paper, although this number included pas only partially based on citizen science, only partially on rare or threatened species, and sometimes on topics other than conservation and monitoring (e.g., a study where citizen scientists provide validated biodiversity data on frogs of Australia, another monitoring humpback whale [*Megaptera novaeangliae*] behavior). Theobald et al. (2015) found a similar proportion for biodiversity citizen science projects: 12% of environmental citizen science projects were recorded as leading to a peer‐reviewed publication based on traditional literature‐based methods compared with 45−60% based on a manager‐survey method. Theobald et al. (2015) propose that the inconsistent responses by project managers to survey questions may be indicative of confusion regarding the meaning of *peer reviewed*, leading to inclusion of gray literature and technical reports in self‐reported publications. Our results do not support this explanation because we specifically asked for 3 different types of contributions and distinguished between gray literature and peer‐reviewed publications. Moreover, most of our respondents provided titles or links to back up the number of contributions they provided. We therefore propose that traditional literature‐based methods underestimate publication rates, most likely due to the lack of direct or prominent mention of the project origins in peer‐reviewed publications.

### Predictors of scientific contribution success

Citizen science is experiencing an explosion in growth, but the use of data produced by citizen science projects in scientific publications and conservation decision‐making has not reached its full potential yet (Newman et al., 2017). Various citizen science projects highlight the importance of several factors that lead to tangible contributions to science and conservation. These include design and implementation strategies that match the needs of science and public engagement with the right type of project and approach to maximize collaboration among stakeholders, from volunteers to decision makers, while integrating cross‐disciplinary approaches (Maas et al., 2021; Newman et al., 2017; Robinson et al., 2018).

Longevity was the most important factor influencing the number of peer‐reviewed papers, gray literature publications, and conservation measures. This result aligns with those of Tulloch et al. (2013) and Theobald et al. (2015), who analyzed peer‐reviewed publications of birds and biodiversity citizen science projects, respectively. Chandler, Rullman, et al. (2017) also found a similar result; the longevity of Earthwatch‐supported projects was highly correlated with publication rate and contributions to management plans. In this study, contributions increased over time, peaking for management plans and policies at 6–8 years, which was earlier than contributions to publications that peaked at 7–9 years. Project implementation is complex and has a strong element of trial and error that involves human, budgetary, and technical constraints. Furthermore, data accumulation takes time, especially for rare or threatened species. As such, long‐term studies may be more likely to lead to scientific contributions because they improve the ability to measure change over space and time (Bird et al., 2014; Tulloch et al., 2013).

The success of long‐term projects may be related to several factors, such as co‐funding to maintain a long‐term financial support (Conrad & Hilchey, 2011; Cunha et al., 2017) and methods that reflect the goal of the project and the motivations and capabilities of the target volunteer profile, which avoids bias in the data set and loss of motivation due to frustration (Chandler, Rullman, et al., 2017; Couvet et al., 2008; McKinley et al., 2017). Multiple organization types leading a project (e.g., university + NGOs) bring a range of scientists and citizen scientists together and may provide 2 major elements that are often lacking in projects run by a single organization: recruitment with long‐term engagement and scientific rigor (Cunha et al., 2017).

Data quality was another important predictor of success in terms of peer‐reviewed and gray literature publications, and to a lesser extent, conservation measures. This result differs from Theobald et al.’s (2015) results that probability of publication was largely unaffected by the data quality assurance measures. This difference with our finding may mean that an index combining the most important metrics may be a better measure of data quality than individual metrics, either because the combined effect is greater than the sum of their separate effects or because the variance in our sample was bigger than the earlier study. Data quality is particularly relevant when studying rare and threatened species because misidentification of threatened animals could have broader legal implications (Ely et al., 2017), than, for instance, biodiversity. Data sets produced by citizen scientists using rigorous methods can reliably produce high‐quality outputs on par with those produced by professionals (Kosmala et al., 2016; McKinley et al., 2017). A comment from a project manager reflects this: “One of our guiding principles is Do ‐ Discover – Influence: Do good survey work, Discover through data analysis, and Influence policy, conservation management, research priorities, and development assessment processes.”

Our data quality point system, based on a list of important criteria, such as data validation and metadata inclusion, provides a tool to quantify and compare data quality, which will help managers optimize their scientific productivity (see Appendices S12–16 for data quality's predictor of success). However, simpler opportunistic citizen science projects that might have a low data quality index can still provide relevant data on rare species than structured citizen science projects due to higher spatial and temporal sampling coverage, leading to higher detection probabilities (Henckel et al., 2020).

Collaboration type influenced the number of gray literature publications and conservation measures; transdisciplinary projects produced on average more contributions than other approaches. The idea that more holistic projects yield more conservation outcomes is also supported by a study that examined the explicit inclusion of multiple dimensions of place in citizen science project (Newman et al., 2017). It is reasonable to say that transdisciplinarity may not be as important to achieve peer‐reviewed publications because the first objective of this type of contribution is often knowledge advancement. In contrast, a transdisciplinary approach may be essential to enact conservation measures for 3 reasons. First, a transdisciplinary approach can better tackle real‐world problems, such as endangered species conservation, that involve nonacademic stakeholders. Second, powerful new knowledge can be created by merging multiple knowledge streams and different value systems. Third, this approach also increases creativity because stakeholders’ different points of view could inspire others and lead to the best solution to a problem (Putra, 2017). Interdisciplinary citizen science projects, which also include a high level of cooperation, are common in the biological domain (Pettibone et al., 2017) and were well represented in our surveyed projects. Transdisciplinary projects were scarcer, meaning that there is an unexploited potential to improve the scientific value of citizen science projects on rare or threatened species. However, this approach takes more time and effort, which emphasizes the importance of proper planning to ensure projects last long and reach their full potential. Because many funding agencies focus on short‐term deliverables, we advise consideration of the increased benefits of long‐term commitment for citizen science projects focusing on rare or threatened species.

Two potential sources of bias in our study are the process of discovering and selecting projects for analysis and the process for obtaining information about each of the selected projects. We expect we undersampled projects in communities speaking languages other than English, Spanish, French, and Chinese for our spatial and temporal trend analysis and projects in communities speaking languages other than English for our scientific contribution analysis; projects without websites (i.e., very local or small scale); projects fitting the definition of citizen science, although not defining themselves as such or projects founded before the late 2000s, when the terms *citizen science* and *participatory science* became popular; completed projects; and projects not focusing on rare or threatened species but producing scientific contributions on rare or endangered species (e.g., eBird, iNaturalist; Pocock et al., 2015).

Although projects like eBird and iNaturalist generate very important data on rare and threatened species, there are many cases in which a more focused approach will be helpful. For example, iNaturalist relies primarily on photographs and audio and has relatively few bat entries. A more targeted approach for rare or threatened bats is thus warranted. Moreover, because *citizen science* is a term more popular in North America, the use of other keywords, such as *participatory science* or *volunteer*, extended our search geographically. We have no way of estimating how these other kinds of projects might differ from our project's subsample. Nonetheless, our search was standardized, repeatable, and, we believe, an informative sample of this type of project and a valuable starting point for future citizen science projects, and it allowed for a systematic analysis of citizen science focusing on rare or threatened animals. In addition, bats were overrepresented due to our specific search for this taxon in English (see note in Appendix S1). However, because the results for bats only did not differ from other taxa (preliminary results), we are confident that including bats separately did not bias our overall results.

We found that citizen science contributes substantially to rare or threatened animal monitoring and conservation in a wide range of ways, from theoretical frameworks in peer‐reviewed publication to applied conservation measures. Results gained from this study, in which we combined web searches and manager surveys, would have been unachievable with a traditional literature‐based method. To our knowledge, we provide the first quantitative analysis of a large spectrum of scientific contributions of citizen science projects focusing on rare or threatened animals on multiple continents. We also provide indicators to inform the future development of citizen science activities and increase scientific contributions. Although we focused on the quantification of scientific contributions and identification of factors increasing contribution numbers, not all contributions are equal in terms of quality and impact for science and conservation. Quantity is not always a guarantor of success, and future research should take into consideration other indices of success, such as the contribution quality (Chandler, Rullman, et al., 2017).

Citizen science is a promising approach to help scientists address gaps, which is essential to ensure effective conservation of rare or threatened species. Several conservation measures emerged from our study. To increase all types of scientific contributions, we call for increased conceptual and methodological development when starting citizen science projects to ensure project persistence, high‐quality data, and transdisciplinary values in research design and outputs by undertaking the following steps. Plan projects so that they are likely to have long life spans, and find multiple sources of funding as a hedge against cuts in resources (Conrad & Hilchey, 2011; Cunha et al., 2017). Educate and train project managers because this can affect the timely delivery of projects (Brown et al. 2007). Ensure good data collection and management by integrating factors included in our data quality index or those described in Kosmala et al. (2016). Include diverse stakeholders in all stages of the project to ensure best practices that link scientific research to conservation interventions.

## Supporting information

Appendix S1. Full list of key words used to search for citizen science project focusing on rare or threatened species.
Appendix S2. Predictors of success type and category used to evaluate factors affecting the number of scientific contributions of citizen science projects focusing on rare or threatened animals.
Appendix S3. Data quality criteria and its scoring system used to create the data quality index in an examination of predictors of success of scientific contributions of citizen science projects focusing on rare or threatened animals.
Appendix S4. Location Tukey pairwise comparison test with 95% family‐wise confidence level. Since Tukey's test is a post‐hoc test, we first fitted a general linear regression model with a quasi‐Poisson distribution and performed an ANOVA on the data. The glm included the frequency of new projects as y variable and the location and the polynomial function of the year to consider the non‐linearity as x variables.
Appendix S5. Predictors of success included in each of the scientific contribution global model of citizen science projects focusing on rare or threatened animals.
Appendix S6. Full model selection of predictors of success on scientific paper publications ranked by their corresponding Akaike's information criterion corrected for small sample size (AICc) of citizen science projects focusing on rare or threatened animals. ΔiAICc is the difference between AICc for the current model and the minimum of AICc among all the models. ωi = Akaike weights.
Appendix S7. Full predictor coefficient values, standard errors, t‐values, p‐values, and confidence intervals of the best model for scientific paper predictors of success of citizen science projects focusing on rare or threatened animals.
Appendix S8. Full model selection of predictors of success on grey literature publications ranked by their corresponding Akaike's information criterion corrected for small sample size (AICc) of citizen science projects focusing on rare or threatened animals. ΔiAICc is the difference between AICc for the current model and the minimum of AICc among all the models. ωi = Akaike weights.
Appendix S9. Full predictor coefficient values, standard errors, t‐values, p‐values, and confidence intervals of the best model for grey literature predictors of success of citizen science projects focusing on rare or threatened animals.
Appendix S10. Full model selection of predictors of success on conservation measures ranked by their corresponding Akaike's information criterion corrected for small sample size (AICc) of citizen science projects focusing on rare or threatened animals. ΔiAICc is the difference between AICc for the current model and the minimum of AICc among all the models. ωi = Akaike weights.
Appendix S11. Full predictor coefficient values, standard errors, t‐values, p‐values, and confidence intervals of the best model for conservation measures predictors of success of citizen science projects focusing on rare or threatened animals.
Appendix S12. Top‐5 of model selection of predictors of success on data quality index of citizen science projects focusing on rare or threatened animals, ranked by their corresponding Akaike's information criterion corrected for small sample size (AICc). ΔiAICc is the difference between AICc for the current model and the minimum of AICc among all the models. ωi = Akaike weights. Data quality index was assessed from the survey by attributing a score based on the following criteria: protocol freely available, support and training, data entry, metadata, verifiability, validation and data standardization (See appendix S3).
Appendix S13. √(1−quality index) as used in the linear model in function of a) methodology complexity, b) organisation type, and c) location of citizen science projects focusing on rare or threatened animals. Data quality index was assessed from the survey by attributing a score based on the following criteria: protocol freely available, support and training, data entry, metadata, verifiability, validation and data standardization (See appendix S3). Low √(1‐quality index) means high data quality and high √(1‐quality index) means low data quality, as assessed by our scoring system, In panel b), Gov means governmental organization, NPO means Non‐profit organization, Acad means Academic organization, CDO means citizen‐driven organization, Ind means Industrial organization, and mixed means more than two organizations as the main leader of the project.
Appendix S14. Tukey pairwise comparison test with 95% family‐wise confidence level for the methodology complexity of citizen science projects focusing on rare or threatened animals. Since Tukey's test is a post‐hoc test, we first fitted the best linear regression model (lm) and performed ANOVA on the data.
Appendix S15. Tukey pairwise comparison test with 95% family‐wise confidence level for the organization type of citizen science projects focusing on rare or threatened animals. Since Tukey's test is a post‐hoc test, we first fitted the best linear regression model (lm) and performed ANOVA on the data. Gov means governmental organization, NPO means Non‐profit organization, Acad means Academic organization, CDO means citizen‐driven organization, Ind means Industrial organization, and mixed means more than 2 organization as the main leader of the project.
Appendix S16. Tukey pairwise comparison test with 95% family‐wise confidence level for the location of citizen science projects focusing on rare or threatened animals. Since Tukey's test is a post‐hoc test, we first fitted the best linear regression model (lm) and performed ANOVA on the data*.
Appendix S17. Survey sent to managers of citizen science projects focusing on rare or threatened animals
