# Supplementary material for: Scientific contributions of citizen science applied to rare or threatened animals
Source: Conserv Biol. 2022 Oct 13;36(6):e13976. doi: 10.1111/cobi.13976 (PMC10092489; doi:10.1111/cobi.13976)
Supplement: Supplementary file 1 — Appendix S1. Full list of key words used to search for citizen science project focusing on rare or threatened species. Appendix S2. Predictors of success type and category used to evaluate factors affecting the number of scientific contributions of citizen science projects focusing on rare or threatened animals. Appendix S3. Data quality criteria and its scoring system used to create the data quality index in an examination of predictors of success of scientific contributions of citizen science projects focusing on rare or threatened animals. Appendix S4. Location Tukey pairwise comparison test with 95% family‐wise confidence level. Since Tukey's test is a post‐hoc test, we first fitted a general linear regression model with a quasi‐Poisson distribution and performed an ANOVA on the data. The glm included the frequency of new projects as y variable and the location and the polynomial function of the year to consider the non‐linearity as x variables. Appendix S5. Predictors of success included in each of the scientific contribution global model of citizen science projects focusing on rare or threatened animals. Appendix S6. Full model selection of predictors of success on scientific paper publications ranked by their corresponding Akaike's information criterion corrected for small sample size (AICc) of citizen science projects focusing on rare or threatened animals. ΔiAICc is the difference between AICc for the current model and the minimum of AICc among all the models. ωi = Akaike weights. Appendix S7. Full predictor coefficient values, standard errors, t‐values, p‐values, and confidence intervals of the best model for scientific paper predictors of success of citizen science projects focusing on rare or threatened animals. Appendix S8. Full model selection of predictors of success on grey literature publications ranked by their corresponding Akaike's information criterion corrected for small sample size (AICc) of citizen science projects focusing on rare or threate [file COBI-36-0-s001.docx]

**Supporting Information**

**Appendix S1.** **Full list of key words used to search for citizen science project focusing on rare or threatened species.**

| *Language* | | *Key words used for web searches* | |  |  |
| --- | --- | --- | --- | --- | --- |
| English | | Species terms | rare, threatened, endangered, bat, chiropter* |  |  |
|  |  | Project type terms | "citizen science", "citizen-based", "public particip*", particip*, volunteer* |  |  |
|  |  | Establishment goal terms | program, project, monitor*, watch |  |  |
|  |  | Location terms | Australia*, Oceania*, Asia*, Europe*, Russia*, Africa*, America*, worldwide, internation* |  |  |
| Spanish | | Species terms | rara*, raro*, amenazad*, peligro, especie, fauna |  |  |
|  |  | Project type terms | “ciencia ciudadana”, particip*, participativo, voluntari* |  |  |
|  |  | Establishment goal terms | (programa or proyecto), observa*, monitor* |  |  |
|  |  | Location terms | Mexico, Ecuador, Peru, “Costa Rica”, España, Colombia, Cuba, Guatemala, Venezuela, Argentina, Panama, Uruguay, Paraguay, Honduras, “Puerto Rico”, Bolivia, Salvador |  |  |
| French | | Species terms | rare*, menacé*, vulnerable*, Tortuga*, anfibio, ballena*, tiburón*, mamifere, ave*, murcielago, insecto |  |  |
|  |  | Project type terms | “science citoyenne”, “science participative”,  (volontaire ou bénévole) |  |  |
|  |  | Establishment goal terms | projet, programme, suivi |  |  |
| Chinese | | Species terms | (受威胁的 or 濒危的), “物种 的” |  |  |
|  |  | Project type terms | “公民科学”, 参与*, 志愿* |  |  |
|  |  | Establishment goal terms | (方案 or 项目 or 监控） |  |  |
|  | | When searching projects, each species term above was searched individually with each project and/or establishment goal and/or location terms. The first 100 links returned were screened to assess if the project fitted the predefined inclusion criteria. | | | |
|  | | *Major citizen science directories included in web searches* | | | |
|  | | scistarter.com, citizenscience.gov (USA), zooniverse.org, volunteer.gov, inaturalist.org, scientificamerican.com, biocollect.ala.org.au, and CitSci.org. | | | |

Note that “bat” and “chiropter*” have been included in key words in the English web search since the study primary aimed to compare bat to other taxa projects. Since the preliminary analysis of bat only projects provided similar results than other taxa projects, we decided to include bat projects in the overall analysis.

**Appendix S2.** **Predictors of success type and category used to evaluate factors affecting the number of scientific contributions of citizen science projects focusing on rare or threatened animals.**

| *Predictors* | *Type* | *Category* |
| --- | --- | --- |
| Longevity (year) | continual | 1945−2019 |
| Location | categorical | North America, Central/South America, Africa, Europe, Russia, Asia, Oceania, Arctic/Antarctica, worldwide |
| Focal taxa | categorical | Mammal, bird, herps (amphibian and reptile), fish, invertebrate (arthropoda, mollusca), mixed |
| Scale of data collection | categorical | Local, regional, national, international |
| Collaboration type | categorical | Intradisciplinary (working within a single discipline. For example, biology), multidisciplinary (people from different disciplines working together, each drawing on their disciplinary knowledge in an additive manner. For example, studying bats from the morphology and disease-carrying point of view), interdisciplinary (integration of knowledge and methods from different disciplines using a synthesis of approaches with stronger level of cooperation), transdisciplinary (Creating a unity of intellectual frameworks beyond the disciplinary perspectives to form a new holistic approach), |
| Organisation type | categorical | Citizen-driven, non-profit, academic, governmental, industrial, mixed of two, mixed (3 organizations or more) |
| Methodological complexity | categorical | Systematic/elaborated monitoring, mixed, mass participation/simple |
| Data quality index | continual | Ranging from 0 to 1 |

**Appendix S3.** **Data quality criteria and its scoring system used to create the data quality index in an examination of predictors of success of scientific contributions of citizen science projects focusing on rare or threatened animals.**

| Factors | Scoring |
| --- | --- |
| Written protocol freely available | yes = 1.5, on request = 1, no = 0 |
| Support and training | in person/personal = 1.5, hard copy or electronic support = 1, none = 0 |
| Data entry | systematic = 1.5, semi-systematic = 1, non-systematic = 0 |
| Metadata | environmental conditions = 0.5, characteristic of volunteers = 0.5, equipment settings (if) =0.5, none = 0 (cumulative) |
| Verifiability | expert = 1.5, photo or audio = 1, optional = 0.5, none = 0 |
| Validation | yes = 1.5, partially = 1, none = 0 |
| Data standardization (if data analysis done) | all = 1.5, some = 1, none = 0, not applicable |

**Appendix S4. Location Tukey pairwise comparison test with 95% family-wise confidence level. Since Tukey’s test is a post-hoc test, we first fitted a general linear regression model with a quasi-Poisson distribution and performed an ANOVA on the data. The glm included the frequency of new projects as y variable and the location and the polynomial function of the year to consider the non-linearity as x variables.**

| *Location pairwise comparisons* | *diff* | *lwr* | *upr* | *p adj* |
| --- | --- | --- | --- | --- |
| America-Africa | −1.216x10^−1^ | −0.690 | 0.446 | 0.999 |
| Antartic-Africa | −1.351x10^−1^ | −0.703 | 0.433 | 0.998 |
| Asia-Africa | −2.702x10^−2^ | −0.595 | 0.541 | 1 |
| Europe-Africa | 2.972x10^−1^ | −0.271 | 0.865 | 0.789 |
| North America-Africa | 1.527 | 0.958 | 2.095 | 0.001x10^−18^* |
| Oceania-Africa | 5.000x10^−1^ | −0.068 | 1.068 | 0.136 |
| South America-Africa | −1.351x10^−1^ | −0.703 | 0.433 | 0.998 |
| Worldwide-Africa | 4.054x10^−2^ | −0.527 | 0.609 | 0.999 |
| Antarctic-America | −1.351x10^−2^ | −0.581 | 0.554 | 1 |
| Asia-America | 9.459x10^−2^ | −0.473 | 0.663 | 0.999 |
| Europe-America | 4.189x10^−1^ | −0.149 | 0.987 | 0.347 |
| North America-America | 1.648 | 1.08 | 2.217 | 0.000x10^−16^* |
| Oceania-America | 6.216x10^−1^ | 0.053 | 1.19 | 0.020* |
| South America-America | −1.351x10^−2^ | −0.581 | 0.554 | 1 |
| Worldwide-America | 1.621x10^−1^ | −0.406 | 0.73 | 0.993 |
| Asia-Antarctic | 1.081x10^−1^ | −0.460 | 0.676 | 0.999 |
| Europe-Antarctic | 4.324x10^−1^ | −0.136 | 1 | 0.303 |
| North America-Antarctic | 1.662 | 1.093 | 2.23 | 0.000x10^−16^* |
| Oceania-Antarctic | 6.351x10^−1^ | 0.066 | 1.203 | 0.016* |
| South America-Antarctic | 2.942x10^−15^ | −0.568 | 0.568 | 1 |
| Worldwide-Antarctic | 1.756x10^−1^ | −0.392 | 0.744 | 0.989 |
| Europe-Asia | 3.243x10^−1^ | −0.244 | 0.892 | 0.698 |
| North America-Asia | 1.554 | 0.985 | 2.122 | 0.000x10^−16^* |
| Oceania-Asia | 5.270x10^−1^ | −0.041 | 1.095 | 0.094 |
| South America-Asia | −1.081x10^−1^ | −0.676 | 0.46 | 0.999 |
| Worldwide-Asia | 6.756x10^−2^ | −0.501 | 0.636 | 0.999 |
| North America-Europe | 1.229 | 0.661 | 1.798 | 0.000x10^−16^* |
| Oceania-Europe | 2.027x10^−1^ | −0.365 | 0.771 | 0.972 |
| South America-Europe | −4.324x10^−1^ | −1.001 | 0.136 | 0.303 |
| Worldwide-Europe | −2.567x10^−1^ | −0.825 | 0.311 | 0.895 |
| Oceania-North America | −1.027 | −1.595 | −0.458 | 1.000x10^−6^* |
| South America-North Amer | −1.662 | −2.230 | −1.093 | 0.000x10^−16^* |
| Worldwide-North America | −1.486 | −2.054 | −0.918 | 0.000x10^−16^* |
| South America-Oceania | −6.351x10^−1^ | −1.203 | −0.066 | 0.016* |
| Worldwide-Oceania | −4.594x10^−1^ | −1.027 | 0.109 | 0.226 |
| Worldwide-South America | 1.756x10^−1^ | −0.392 | 0.744 | 0.989 |

**Appendix S5.** **Predictors of success included in each of the scientific contribution global model of citizen science projects focusing on rare or threatened animals.**

| *Predictors of success* | *Scientific paper* | *Grey literature* | *Conservation measure* | *Data quality index* |
| --- | --- | --- | --- | --- |
| Longevity | x | x | x | x |
| Location | x | x | x | x |
| Scale of data collection | NA | NA | x | NA |
| Taxa | x | x | x | NA |
| Collaboration type | x | x | x | NA |
| Organisation type | x | x | x | x |
| Data quality index | x | x | x | NA |
| Methodological complexity | NA | NA | NA | x |

**Appendix S6.** **Full model selection of predictors of success on scientific paper publications ranked by their corresponding Akaike’s information criterion corrected for small sample size (AICc) of citizen science projects focusing on rare or threatened animals. ΔiAICc is the difference between AICc for the current model and the minimum of AICc among all the models. ωi = Akaike weights.**

| *Models* | *AIC_c_* | *∆ AIC_c_* | *w_i_* |
| --- | --- | --- | --- |
| log(longevity) + √(1−quality index) | 287.5 | 0.00 | 0.689 |
| log(longevity) + √(1−quality index) + location | 290.8 | 3.26 | 0.135 |
| log(longevity) + √(1−quality index) + collaboration | 291.5 | 3.98 | 0.094 |
| log(longevity) + √(1−quality index) + organisation | 294.4 | 6.91 | 0.022 |
| log(longevity) | 294.7 | 7.19 | 0.019 |
| log(longevity) + location | 295.7 | 8.21 | 0.011 |
| log(longevity) + √(1−quality index) + location + collaboration | 296.3 | 8.75 | 0.009 |
| log(longevity) + √(1−quality index) + taxa | 296.4 | 8.85 | 0.008 |
| log(longevity) + collaboration | 297.4 | 9.91 | 0.005 |
| log(longevity) + √(1−quality index) + taxa + collaboration | 299.8 | 12.33 | 0.001 |
| log(longevity) + location + collaboration | 299.9 | 12.42 | 0.001 |
| log(longevity) + √(1−quality index) + collaboration + organisation | 300.1 | 12.55 | 0.001 |
| log(longevity) + √(1−quality index) + location + taxa | 301.0 | 13.51 | 0.001 |
| log(longevity) + √(1−quality index) + location + organisation | 301.1 | 13.61 | 0.001 |
| log(longevity) + taxa | 302.1 | 14.57 | 0.000 |
| log(longevity) + organisation | 303.1 | 15.59 | 0.000 |
| log(longevity) + location + organisation | 304.0 | 16.49 | 0.000 |
| log(longevity) + collaboration + taxa | 304.3 | 16.82 | 0.000 |
| log(longevity) + √(1−quality index) + location + collaboration + taxa | 304.6 | 17.07 | 0.000 |
| √(1-−quality index) | 305.2 | 17.73 | 0.000 |
| log(longevity) + location + taxa | 305.5 | 18.04 | 0.000 |
| log(longevity) + √(1−quality index) + location + taxa | 305.9 | 18.36 | 0.000 |
| log(longevity) + location + collaboration | 307.9 | 20.36 | 0.000 |
| log(longevity) + collaboration + organisation | 308.0 | 20.54 | 0.000 |
| log(longevity) + √(1−quality index) + location + collaboration + organisation | 308.1 | 20.64 | 0.000 |
| √(1−quality index) + taxa | 309.4 | 21.88 | 0.000 |
| √(1−quality index) + collaboration | 310.0 | 22.50 | 0.000 |
| √(1−quality index) + organisation | 310.3 | 22.84 | 0.000 |
| log(longevity) + location + collaboration + organisation | 310.4 | 22.89 | 0.000 |
| √(1−quality index) + location | 311.3 | 23.80 | 0.000 |
| log(longevity) + √(1−quality index) + organisation + taxa | 312.2 | 24.67 | 0.000 |
| log(longevity) + organisation + taxa | 312.8 | 25.34 | 0.000 |
| null model | 313.4 | 25.91 | 0.000 |
| √(1−quality index) + collaboration + taxa | 314.4 | 26.85 | 0.000 |
| log(longevity) + √(1−quality index)+ location + organisation + taxa | 314.4 | 26.86 | 0.000 |
| √(1−quality index) + organisation | 315.4 | 27.93 | 0.000 |
| √(1−quality index) + organisation + taxa | 316.9 | 29.43 | 0.000 |
| √(1−quality index) + location + taxa | 316.9 | 29.43 | 0.000 |
| location | 316.9 | 29.43 | 0.000 |
| taxa | 317.0 | 29.48 | 0.000 |
| log(longevity) + location + organisation + taxa | 317.2 | 29.66 | 0.000 |
| √(1−quality index) + location + collaboration | 317.2 | 29.66 | 0.000 |
| collaboration | 317.5 | 29.97 | 0.000 |
| √(1−quality index) + location + organisation | 318.0 | 30.46 | 0.000 |
| organisation | 318.8 | 31.31 | 0.000 |
| log(longevity) + collaboration + organisation + taxa | 319.2 | 31.73 | 0.000 |
| log(longevity) + √(1−quality index) + location + collaboration + organisation + taxa | 320.5 | 33.01 | 0.000 |
| location + organisation | 320.9 | 33.43 | 0.000 |
| collaboration + taxa | 321.1 | 33.63 | 0.000 |
| location + taxa | 321.6 | 34.10 | 0.000 |
| location + collaboration | 321.6 | 34.12 | 0.000 |
| √(1−quality index) + location + collaboration + taxa | 322.3 | 34.85 | 0.000 |
| √(1−quality index) + collaboration + organisation + taxa | 323.1 | 35.59 | 0.000 |
| collaboration + organisation | 323.5 | 35.99 | 0.000 |
| log(longevity) + location + collaboration + organisation + taxa | 323.7 | 36.22 | 0.000 |
| √(1−quality index) + location + collaboration + organisation | 323.8 | 36.27 | 0.000 |
| organisation + taxa | 324.9 | 37.42 | 0.000 |
| location + collaboration + organisation | 325.3 | 37.83 | 0.000 |
| √(1−quality index) + location + organisation + taxa | 325.8 | 38.34 | 0.000 |
| location + collaboration + taxa | 326.0 | 38.50 | 0.000 |
| location + taxa | 328.0 | 40.52 | 0.000 |
| collaboration + organisation + taxa | 331.5 | 43.99 | 0.000 |
| √(1−quality index) + location + collaboration + organisation + taxa | 332.7 | 45.24 | 0.000 |
| location + collaboration + organisation + taxa | 334.6 | 47.10 | 0.000 |

**Appendix S7.** **Full predictor coefficient values, standard errors, t-values, p-values, and confidence intervals of the best model for scientific paper predictors of success of citizen science projects focusing on rare or threatened animals.**

| *Predictors* | *Value* | *Std. Error* | *t value* | *p value* | *CI upper* | *CI lower* |
| --- | --- | --- | --- | --- | --- | --- |
| log(longevity) | 1.040 | 0.245 | 4.242 | 2.21x10^−5^ | 0.573 | 1.538 |
| √(1−quality index) | −3.905 | 1.344 | −2.904 | 3.67x10^−3^ | −6.669 | −1.369 |
| sc paper: int 1 | 0.368 | 0.861 | 0.427 | 6.69x10^−1^ | Na | Na |
| sc paper: int 2-5 | 0.944 | 0.861 | 1.096 | 2.73x10^−1^ | Na | Na |
| sc paper: int 6-10 | 2.095 | 0.877 | 2.386 | 1.69x10^−2^ | Na | Na |
| sc paper: int >10 | 2.653 | 0.896 | 2.959 | 3.08x10^−3^ | Na | Na |

**Appendix S8.** **Full model selection of predictors of success on grey literature publications ranked by their corresponding Akaike’s information criterion corrected for small sample size (AICc) of citizen science projects focusing on rare or threatened animals. ΔiAICc is the difference between AICc for the current model and the minimum of AICc among all the models. ωi = Akaike weights.**

| *Models* | *AIC_c_* | *∆ AIC_c_* | *w_i_* |
| --- | --- | --- | --- |
| log(longevity) + √(1−quality index) + collaboration | 331.7 | 0.00 | 0.584 |
| log(longevity) + √(1−quality index) | 333.9 | 2.15 | 0.199 |
| log(longevity) + collaboration | 334.5 | 2.76 | 0.147 |
| log(longevity) | 337.1 | 5.41 | 0.039 |
| log(longevity) + √(1−quality index) + collaboration + taxa | 340.0 | 8.28 | 0.009 |
| log(longevity) + √(1−quality index) + location + collaboration | 340.5 | 8.73 | 0.007 |
| log(longevity) + location + collaboration | 340.7 | 8.93 | 0.007 |
| log(longevity) + √(1−quality index) + location | 342.8 | 11.11 | 0.002 |
| log(longevity) + √(1-quality index) + taxa | 343.5 | 11.80 | 0.002 |
| log(longevity) + location | 344.0 | 12.27 | 0.001 |
| log(longevity) + collaboration + taxa | 344.2 | 12.45 | 0.001 |
| √(1−quality index) + collaboration | 346.1 | 14.33 | 0.000 |
| log(longevity) + taxa | 347.5 | 15.73 | 0.000 |
| √(1−quality index) | 348.1 | 16.35 | 0.000 |
| √(1−quality index) + collaboration + taxa | 348.4 | 16.72 | 0.000 |
| collaboration | 350.1 | 18.35 | 0.000 |
| log(longevity) + √(1−quality index) + location + collaboration + taxa | 350.6 | 18.91 | 0.000 |
| log(longevity) + √(1−quality index) + collaboration + organisation | 351.6 | 19.89 | 0.000 |
| log(longevity) + location + collaboration + taxa | 352.0 | 20.28 | 0.000 |
| null model | 352.9 | 21.14 | 0.000 |
| √(1−quality index) + taxa | 353.0 | 21.31 | 0.000 |
| log(longevity) + √(1−quality index) + organisation | 353.3 | 21.59 | 0.000 |
| log(longevity) + collaboration + organisation | 353.4 | 21.63 | 0.000 |
| log(longevity) + √(1−quality index) + location + organisation + taxa | 354.3 | 22.53 | 0.000 |
| collaboration + taxa | 354.5 | 22.82 | 0.000 |
| log(longevity) + organisation | 355.2 | 23.46 | 0.000 |
| log(longevity) + location + taxa | 356.1 | 24.36 | 0.000 |
| √(1−quality index) + location + collaboration | 356.8 | 25.07 | 0.000 |
| location + collaboration | 358.0 | 26.24 | 0.000 |
| taxa | 358.8 | 27.08 | 0.000 |
| √(1−quality index) + location | 358.9 | 27.18 | 0.000 |
| log(longevity) + √(1−quality index) + collaboration + organisation + taxa | 360.0 | 28.29 | 0.000 |
| log(longevity) + location + collaboration + organisation | 361.4 | 29.63 | 0.000 |
| location | 361.4 | 29.64 | 0.000 |
| √(1−quality index) + location + collaboration + taxa | 361.5 | 29.76 | 0.000 |
| log(longevity) + √(1quality index) + collaboration + organisation | 362.9 | 31.17 | 0.000 |
| √(1-−quality index) + collaboration + organisation | 363.7 | 31.93 | 0.000 |
| location + collaboration + taxa | 364.1 | 32.35 | 0.000 |
| log(longevity) + √(1−quality index) + organisation + taxa | 364.1 | 32.42 | 0.000 |
| log(longevity) + location + organisation | 364.3 | 32.56 | 0.000 |
| log(longevity) + collaboration + organisation + taxa | 364.4 | 32.66 | 0.000 |
| √(1−quality index) + location + organisation | 365.1 | 33.40 | 0.000 |
| √(1−quality index) + organisation | 365.5 | 33.74 | 0.000 |
| √(1-quality index) + collaboration + organisation + taxa | 365.5 | 33.77 | 0.000 |
| √(1−quality index) + location + taxa | 366.2 | 34.49 | 0.000 |
| log(longevity) + organisation + taxa | 366.8 | 35.09 | 0.000 |
| collaboration + organisation | 367.2 | 35.47 | 0.000 |
| organisation | 369.1 | 37.34 | 0.000 |
| location + taxa | 369.2 | 37.46 | 0.000 |
| √(1−quality index) + organisation + taxa | 371.7 | 39.96 | 0.000 |
| collaboration + organisation + taxa | 372.5 | 40.74 | 0.000 |
| location + collaboration + organisation + taxa | 375.0 | 43.30 | 0.000 |
| log(longevity) + √(1−quality index) + location + collaboration + organisation + taxa | 375.5 | 43.78 | 0.000 |
| √(1−quality index) + location + collaboration + organisation | 375.6 | 43.83 | 0.000 |
| log(longevity) + location + collaboration + organisation + taxa | 375.7 | 44.01 | 0.000 |
| organisation + taxa | 376.2 | 44.50 | 0.000 |
| √(1−quality index) + location + organisation | 377.9 | 46.18 | 0.000 |
| location + organisation | 378.5 | 46.74 | 0.000 |
| log(longevity) + location + organisation + taxa | 379.1 | 47.33 | 0.000 |
| log(longevity) + √(1−quality index) + location + organisation + taxa | 379.2 | 47.44 | 0.000 |
| √(1−quality index) + location + collaboration + organisation + taxa | 382.1 | 50.39 | 0.000 |
| location + collaboration + organisation + taxa | 383.3 | 51.61 | 0.000 |
| √(1−quality index) + location + organisation + taxa | 387.6 | 55.84 | 0.000 |
| location + organisation + taxa | 388.2 | 56.52 | 0.000 |

**Appendix S9.** **Full predictor coefficient values, standard errors, t-values, p-values, and confidence intervals of the best model for grey literature predictors of success of citizen science projects focusing on rare or threatened animals.**

| *Predictors* | *Value* | *Std. Error* | *t value* | *p value* | *CI upper* | *CI lower* |
| --- | --- | --- | --- | --- | --- | --- |
| log(longevity) | 0.9660 | 0.245 | 3.935 | 8.31x10^−5^ | 0.495 | 1.461 |
| √(1−quality index) | −2.782 | 1.259 | −2.208 | 0.027 | −5.324 | −0.366 |
| collab: intradisciplinary | −1.012 | 0.418 | −2.417 | 0.015 | −1.846 | −0.191 |
| collab: multidisciplinary | −0.527 | 0.461 | −1.143 | 0.252 | −1.441 | 0.374 |
| collab: transdisciplinary | 0.838 | 0.762 | 1.098 | 0.271 | −0.673 | 2.359 |
| grey lit: int 1 | −0.875 | 0.906 | −0.966 | 0.333 | Na | Na |
| grey lit: int 2-5 | −0.270 | 0.898 | −0.301 | 0.763 | Na | Na |
| grey lit: int 6-10 | 1.233 | 0.917 | 1.343 | 0.179 | Na | Na |
| grey lit: int >10 | 1.728 | 0.931 | 1.855 | 0.063 | Na | Na |

**Appendix S10.** **Full model selection of predictors of success on conservation measures ranked by their corresponding Akaike’s information criterion corrected for small sample size (AICc) of citizen science projects focusing on rare or threatened animals. ΔiAICc is the difference between AICc for the current model and the minimum of AICc among all the models. ωi = Akaike weights.**

| *Models* | *AIC_c_* | *∆ AIC_c_* | *w_i_* |
| --- | --- | --- | --- |
| log(longevity) + √(1−quality index) + collaboration + taxa | 318.2 | 0.00 | 0.183 |
| log(longevity) + collaboration + taxa | 318.8 | 0.52 | 0.141 |
| log(longevity) + √(1−quality index) + taxa | 319.0 | 0.78 | 0.124 |
| log(longevity) + collaboration | 319.8 | 1.52 | 0.085 |
| log(longevity) + √(1−quality index) + collaboration | 319.9 | 1.69 | 0.079 |
| log(longevity) + collaboration + taxa + scale | 320.4 | 2.13 | 0.063 |
| log(longevity) + √(1−quality index) + collaboration + taxa + scale | 320.5 | 2.22 | 0.060 |
| log(longevity) + √(1−quality index) | 320.5 | 2.27 | 0.059 |
| log(longevity) + √(1−quality index) + taxa + scale | 321.4 | 3.13 | 0.038 |
| log(longevity) + taxa | 321.5 | 3.28 | 0.036 |
| log(longevity) | 322.0 | 3.80 | 0.027 |
| log(longevity) + collaboration + scale | 322.3 | 4.08 | 0.024 |
| log(longevity) + √(1−quality index) + collaboration + scale | 323.2 | 4.96 | 0.015 |
| log(longevity) + taxa + scale | 323.2 | 4.96 | 0.015 |
| log(longevity) + √(1−quality index) + scale | 323.9 | 5.70 | 0.011 |
| log(longevity) + collaboration + location | 324.0 | 5.81 | 0.010 |
| log(longevity) + scale | 324.7 | 6.45 | 0.007 |
| log(longevity) + location | 325.5 | 7.30 | 0.005 |
| log(longevity) + collaboration + location + taxa | 326.2 | 8.01 | 0.003 |
| log(longevity) + √(1−quality index) + collaboration + location | 326.3 | 8.12 | 0.003 |
| log(longevity) + √(1−quality index) + location | 326.9 | 8.69 | 0.002 |
| log(longevity) + √(1−quality index) + collaboration + organisation | 327.7 | 9.44 | 0.002 |
| log(longevity) + √(1−quality index) + collaboration + location + taxa | 328.2 | 9.94 | 0.001 |
| log(longevity) + location + taxa | 328.7 | 10.48 | 0.001 |
| log(longevity) + collaboration + organisation | 328.8 | 10.59 | 0.001 |
| log(longevity) + √(1−quality index) + collaboration + organisation + taxa | 328.9 | 10.71 | 0.001 |
| location + taxa | 329.3 | 11.04 | 0.001 |
| log(longevity) + collaboration + location + scale | 330.3 | 12.06 | 0.000 |
| organisation | 331.2 | 12.96 | 0.000 |
| √(1−quality index) + taxa | 331.5 | 13.22 | 0.000 |
| log(longevity) + location + scale | 331.7 | 13.49 | 0.000 |
| log(longevity) + collaboration + location + taxa + scale | 331.8 | 13.58 | 0.000 |
| location + scale | 332.7 | 14.48 | 0.000 |
| √(1−quality index) + collaboration + taxa | 332.9 | 14.65 | 0.000 |
| log(longevity) + √(1−quality index) + organisation + taxa | 333.1 | 14.86 | 0.000 |
| log(longevity) + √(1−quality index) + location + scale | 333.2 | 14.96 | 0.000 |
| √(1−quality index) | 333.6 | 15.36 | 0.000 |
| log(longevity) + √(1−quality index) + collaboration + location + taxa + scale | 333.7 | 15.51 | 0.000 |
| log(longevity) + collaboration + organisation + taxa | 334.3 | 16.03 | 0.000 |
| log(longevity) + location + taxa + scale | 334.4 | 16.20 | 0.000 |
| log(longevity) + organisation | 334.5 | 16.26 | 0.000 |
| log(longevity) + collaboration + organisation + scale | 334.5 | 16.30 | 0.000 |
| √(1−quality index) + taxa + scale | 334.6 | 16.41 | 0.000 |
| log(longevity) + √(1−quality index) + collaboration + organisation + scale | 334.8 | 16.58 | 0.000 |
| taxa | 334.8 | 16.58 | 0.000 |
| collaboration + taxa | 334.9 | 16.64 | 0.000 |
| log(longevity) + √(1−quality index) + location + taxa + scale | 334.9 | 16.67 | 0.000 |
| √(1−quality index) + collaboration | 335.4 | 17.13 | 0.000 |
| √(1−quality index) + collaboration + taxa + scale | 336.2 | 17.94 | 0.000 |
| log(longevity) + √(1−quality index) + collaboration + organisation + taxa + scale | 336.5 | 18.28 | 0.000 |
| collaboration | 337.0 | 18.77 | 0.000 |
| log(longevity) + collaboration + location + organisation | 337.1 | 18.92 | 0.000 |
| taxa + scale | 337.3 | 19.12 | 0.000 |
| collaboration + taxa + scale | 337.5 | 19.27 | 0.000 |
| √(1−quality index) + scale | 337.9 | 19.63 | 0.000 |
| log(longevity) + √(1−quality index) + organisation + scale | 337.9 | 19.69 | 0.000 |
| log(longevity) + √(1−quality index) + collaboration + location + organisation | 339.1 | 20.84 | 0.000 |
| √(1−quality index) + collaboration + scale | 339.8 | 21.53 | 0.000 |
| log(longevity) + organisation + scale | 339.8 | 21.58 | 0.000 |
| log(longevity) + organisation + taxa | 339.9 | 21.65 | 0.000 |
| log(longevity) + √(1−quality index) + organisation + taxa + scale | 340.0 | 21.72 | 0.000 |
| scale | 340.2 | 21.92 | 0.000 |
| log(longevity) + collaboration + organisation + taxa + scale | 340.2 | 21.99 | 0.000 |
| collaboration + scale | 340.8 | 22.53 | 0.000 |
| log(longevity) + location + organisation | 341.6 | 23.37 | 0.000 |
| log(longevity) + √(1−quality index) + location + organisation | 342.3 | 24.09 | 0.000 |
| √(1−quality index) + location | 343.1 | 24.87 | 0.000 |
| collaboration + location | 343.1 | 24.88 | 0.000 |
| location | 343.3 | 25.08 | 0.000 |
| √(1−quality index) + location + taxa | 343.5 | 25.29 | 0.000 |
| collaboration + location + taxa | 343.5 | 25.30 | 0.000 |
| log(longevity) + √(1−quality index) + collaboration + location + organisation + taxa | 343.6 | 25.37 | 0.000 |
| location + taxa | 343.9 | 25.65 | 0.000 |
| √(1−quality index) + collaboration + location | 344.1 | 25.87 | 0.000 |
| √(1−quality index) + collaboration + location + taxa | 344.5 | 26.27 | 0.000 |

**Appendix S11.** **Full predictor coefficient values, standard errors, t-values, p-values, and confidence intervals of the best model for conservation measures predictors of success of citizen science projects focusing on rare or threatened animals.**

| *Predictors* | *Value* | *Std. Error* | *t value* | *p value* | *CI upper* | *CI lower* |
| --- | --- | --- | --- | --- | --- | --- |
| log(longevity) | 1.035 | 0.246 | 4.204 | 2.62x10^−5^ | 0.565 | 1.535 |
| collab: intradisciplinary | −0.309 | 0.415 | −0.745 | 4.56x10^−1^ | −1.127 | 0.504 |
| collab: multidisciplinary | 0.230 | 0.476 | 0.482 | 6.29x10^−1^ | −0.709 | 1.167 |
| collab: transdisciplinary | 2.040 | 0.800 | 2.548 | 1.08x10^−2^ | 0.502 | 3.692 |
| cons mes: int 1 | 1.666 | 0.614 | 2.711 | 6.70x10^−3^ | Na | Na |
| cons mes: int 2-5 | 2.458 | 0.634 | 3.875 | 1.06x10^−4^ | Na | Na |
| cons mes: int 6-10 | 3.952 | 0.697 | 5.666 | 1.45x10^−8^ | Na | Na |
| cons mes: int >10 | 4.410 | 0.722 | 6.106 | 1.01x10^−9^ | Na | Na |

**Data quality analysis results**

**Appendix S12.** **Top-5 of model selection of predictors of success on data quality index of citizen science projects focusing on rare or threatened animals, ranked by their corresponding Akaike’s information criterion corrected for small sample size (AICc). ΔiAICc is the difference between AICc for the current model and the minimum of AICc among all the models. ωi = Akaike weights.** **Data quality index was assessed from the survey by attributing a score based on the following criteria: protocol freely available, support and training, data entry, metadata, verifiability, validation and data standardization (See appendix S3).**

| *Models* | *AIC_c_* | *∆ AIC_c_* | *w_i_* |
| --- | --- | --- | --- |
| 1. method complexity + organisation + location | −142.5 | 0.00 | 0.472 |
| 2. method complexity + organisation + location + longevity | −141.0 | 1.46 | 0.227 |
| 3. method complexity + organisation | −140.1 | 2.37 | 0.145 |
| 4. method complexity + organisation + location | −138.2 | 4.27 | 0.056 |
| 5. method complexity + organisation + location + collaboration | −136.7 | 5.85 | 0.025 |


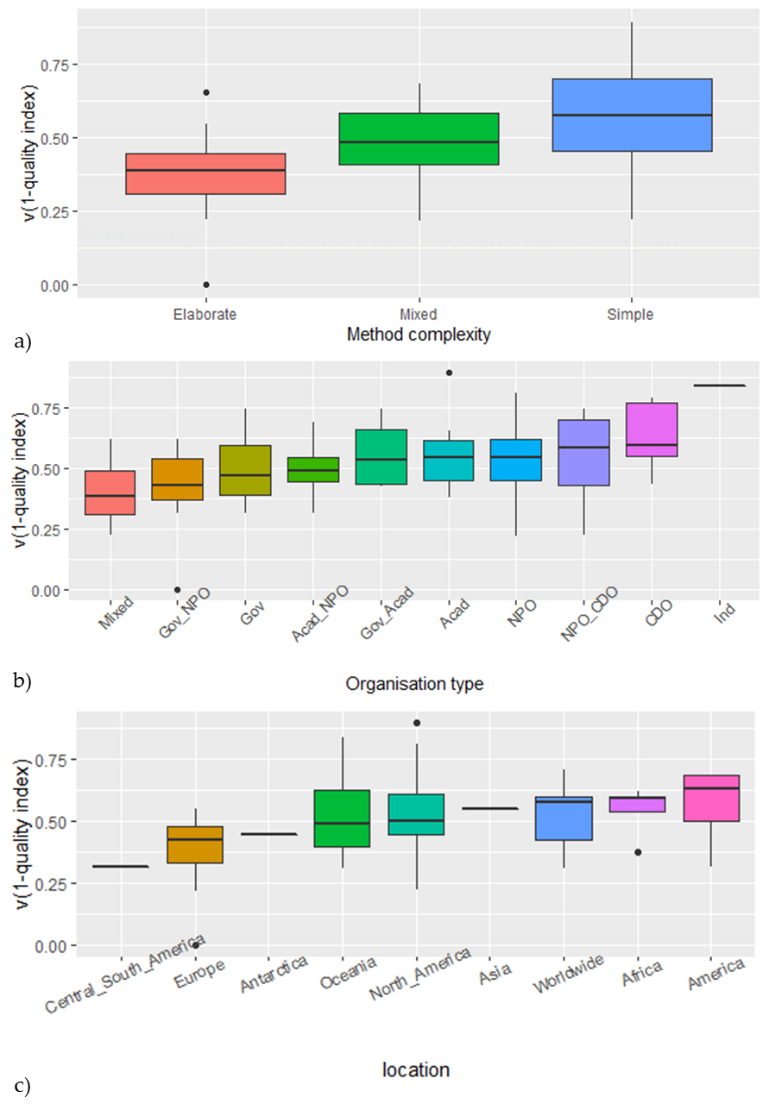


**Appendix S13.** **√(1−quality index) as used in the linear model in function of a) methodology complexity, b) organisation type, and c) location of citizen science projects focusing on rare or threatened animals. Data quality index was assessed from the survey by attributing a score based on the following criteria: protocol freely available, support and training, data entry, metadata, verifiability, validation and data standardization (See appendix S3). Low √(1-quality index) means high data quality and high √(1-quality index) means low data quality, as assessed by our scoring system, In panel b), Gov means governmental organization, NPO means Non-profit organization, Acad means Academic organization, CDO means citizen-driven organization, Ind means Industrial organization, and mixed means more than two organizations as the main leader of the project.**

**Appendix S14.** **Tukey pairwise comparison test with 95% family-wise confidence level for the methodology complexity of citizen science projects focusing on rare or threatened animals. Since Tukey’s test is a post-hoc test, we first fitted the best linear regression model (lm) and performed ANOVA on the data.**

| *Pairewise comparisons* | *diff* | *lwr* | *upr* | *p adj* |
| --- | --- | --- | --- | --- |
| Mixed-Elaborate | 0.095 | 0.016 | 0.173 | 0.013 |
| Simple-Elaborate | 0.182 | 0.106 | 0.257 | 3.00x10^-7^ |
| Simple-Mixed | 0.086 | 0.030 | 0.143 | 0.001 |

**Appendix S15.** **Tukey pairwise comparison test with 95% family-wise confidence level for the organization type of citizen science projects focusing on rare or threatened animals. Since Tukey’s test is a post-hoc test, we first fitted the best linear regression model (lm) and performed ANOVA on the data. Gov means governmental organization, NPO means Non-profit organization, Acad means Academic organization, CDO means citizen-driven organization, Ind means Industrial organization, and mixed means more than 2 organization as the main leader of the project.**

| *Pairewise comparisons* | *diff* | *lwr* | *upr* | *p adj* |
| --- | --- | --- | --- | --- |
| Mixed-Gov_NPO | 0.020 | −0.147 | 0.189 | 0.999 |
| Acad_NPO-Gov_NPO | 0.081 | −0.115 | 0.277 | 0.943 |
| Gov-Gov_NPO | 0.090 | −0.069 | 0.251 | 0.711 |
| NPO_CDO-Gov_NPO | 0.106 | −0.098 | 0.312 | 0.801 |
| Gov_Acad-Gov_NPO | 0.109 | −0.123 | 0.342 | 0.879 |
| Acad-Gov_NPO | 0.127 | −0.039 | 0.293 | 0.297 |
| NPO-Gov_NPO | 0.149 | 0.002 | 0.296 | 0.044* |
| CDO-Gov_NPO | 0.194 | −0.021 | 0.411 | 0.116 |
| Ind-Gov_NPO | 0.380 | −0.022 | 0.783 | 0.081 |
| Acad_NPO-Mixed | 0.060 | −0.115 | 0.236 | 0.982 |
| Gov-Mixed | 0.070 | −0.063 | 0.203 | 0.796 |
| NPO_CDO-Mixed | 0.086 | −0.099 | 0.271 | 0.888 |
| Gov_Acad-Mixed | 0.088 | −0.126 | 0.304 | 0.943 |
| Acad-Mixed | 0.106 | −0.035 | 0.247 | 0.317 |
| NPO-Mixed | 0.128 | 0.010 | 0.246 | 0.021* |
| CDO-Mixed | 0.174 | −0.024 | 0.372 | 0.136 |
| Ind-Mixed | 0.359 | −0.033 | 0.753 | 0.103 |
| Gov-Acad_NPO | 0.009 | −0.158 | 0.177 | 1.000 |
| NPO_CDO-Acad_NPO | 0.025 | −0.185 | 0.237 | 0.999 |
| Gov_Acad-Acad_NPO | 0.028 | −0.209 | 0.266 | 0.999 |
| Acad-Acad_NPO | 0.046 | −0.128 | 0.219 | 0.997 |
| NPO-Acad_NPO | 0.068 | −0.087 | 0.223 | 0.918 |
| CDO-Acad_NPO | 0.113 | −0.108 | 0.336 | 0.817 |
| Ind-Acad_NPO | 0.299 | −0.106 | 0.705 | 0.345 |
| NPO_CDO-Gov | 0.016 | −0.162 | 0.193 | 0.999 |
| Gov_Acad-Gov | 0.018 | −0.190 | 0.227 | 0.999 |
| Acad-Gov | 0.036 | −0.095 | 0.167 | 0.996 |
| NPO-Gov | 0.058 | −0.047 | 0.164 | 0.742 |
| CDO-Gov | 0.104 | −0.087 | 0.295 | 0.757 |
| Ind-Gov | 0.289 | −0.100 | 0.679 | 0.334 |
| Gov_Acad-NPO_CDO | 0.002 | −0.242 | 0.248 | 1.000 |
| Acad-NPO_CDO | 0.020 | −0.163 | 0.203 | 0.999 |
| NPO-NPO_CDO | 0.042 | −0.123 | 0.208 | 0.998 |
| CDO-NPO_CDO | 0.088 | −0.142 | 0.318 | 0.964 |
| Ind-NPO_CDO | 0.273 | −0.136 | 0.684 | 0.492 |
| Acad-Gov_Acad | 0.017 | −0.196 | 0.231 | 0.999 |
| NPO-Gov_Acad | 0.039 | −0.159 | 0.239 | 0.999 |
| CDO-Gov_Acad | 0.085 | −0.169 | 0.340 | 0.985 |
| Ind-Gov_Acad | 0.270 | −0.154 | 0.695 | 0.556 |
| NPO-Acad | 0.022 | −0.092 | 0.137 | 0.999 |
| CDO-Acad | 0.067 | −0.128 | 0.264 | 0.981 |
| Ind-Acad | 0.253 | −0.139 | 0.645 | 0.538 |
| CDO-NPO | 0.045 | −0.134 | 0.225 | 0.998 |
| Ind-NPO | 0.231 | −0.153 | 0.615 | 0.638 |
| Ind-CDO | 0.185 | −0.231 | 0.601 | 0.910 |

**Appendix S16.** **Tukey pairwise comparison test with 95% family-wise confidence level for the location of citizen science projects focusing on rare or threatened animals. Since Tukey’s test is a post-hoc test, we first fitted the best linear regression model (lm) and performed ANOVA on the data*.**

| *Pairewise comparisons* | *diff* | *lwr* | *upr* | *p adj* |
| --- | --- | --- | --- | --- |
| Europe-Central_South_America | 0.058 | −0.331 | 0.449 | 0.999 |
| Antarctica-Central_South_America | 0.130 | −0.395 | 0.657 | 0.996 |
| Oceania-Central_South_America | 0.197 | −0.183 | 0.578 | 0.777 |
| Worldwide-Central_South_America | 0.208 | −0.186 | 0.603 | 0.761 |
| North_America-Central_South_America | 0.218 | −0.156 | 0.593 | 0.651 |
| Africa-Central_South_America | 0.228 | −0.187 | 0.644 | 0.720 |
| Asia-Central_South_America | 0.231 | −0.295 | 0.758 | 0.897 |
| America-Central_South_America | 0.247 | −0.159 | 0.655 | 0.596 |
| Antarctica-Europe | 0.072 | −0.318 | 0.462 | 0.999 |
| Oceania-Europe | 0.138 | −0.003 | 0.280 | 0.060. |
| Worldwide-Europe | 0.149 | −0.027 | 0.326 | 0.166 |
| North_America-Europe | 0.159 | 0.033 | 0.285 | 0.003* |
| Africa-Europe | 0.169 | −0.050 | 0.389 | 0.273 |
| Asia-Europe | 0.172 | −0.217 | 0.563 | 0.893 |
| America-Europe | 0.189 | −0.014 | 0.393 | 0.091 |
| Oceania-Antarctica | 0.066 | −0.314 | 0.447 | 0.999 |
| Worldwide-Antarctica | 0.077 | −0.317 | 0.472 | 0.999 |
| North_America-Antarctica | 0.087 | −0.287 | 0.462 | 0.998 |
| Africa-Antarctica | 0.097 | −0.318 | 0.513 | 0.998 |
| Asia-Antarctica | 0.100 | −0.426 | 0.627 | 0.999 |
| America-Antarctica | 0.116 | −0.290 | 0.524 | 0.991 |
| Worldwide-Oceania | 0.010 | −0.142 | 0.164 | 0.999 |
| North_America-Oceania | 0.020 | −0.070 | 0.112 | 0.998 |
| Africa-Oceania | 0.030 | −0.171 | 0.233 | 0.999 |
| Asia-Oceania | 0.033 | −0.346 | 0.414 | 0.999 |
| America-Oceania | 0.050 | −0.134 | 0.234 | 0.994 |
| North_America-Worldwide | 0.010 | −0.129 | 0.149 | 0.999 |
| Africa-Worldwide | 0.020 | −0.207 | 0.248 | 0.999 |
| Asia-Worldwide | 0.023 | −0.371 | 0.418 | 1.000 |
| America-Worldwide | 0.039 | −0.172 | 0.251 | 0.999 |
| Africa-North_America | 0.009 | −0.181 | 0.201 | 1.000 |
| Asia-North_America | 0.013 | −0.362 | 0.388 | 1.000 |
| America-North_America | 0.029 | −0.143 | 0.202 | 0.999 |
| Asia-Africa | 0.003 | −0.413 | 0.419 | 1.000 |
| America-Africa | 0.019 | −0.230 | 0.269 | 0.999 |
| America-Asia | 0.016 | −0.391 | 0.424 | 1.000 |

* We also tried to test the influence of predictors on database size, but different projects had different meaningful unit to evaluate the database size (i.e. number of sites, count, and observations). As the subsample created by those three divisions was too low for the global model to converge, we were unable to evaluate the influence of predictors on the database size.

**Appendix S17.** **Survey sent to managers of citizen science projects focusing on rare or threatened animals**

**Scientific contributions of citizen science projects focusing on rare or threatened species.**

**___________________________________________________**

1. **Project name:**

**_____________________________________________________________________**

1. **How many years have you been (or were) managing this program?**

- < 1 year
- 1-2 years
- 2-5 years
- ≥ 5 years

1. **What is your highest level of educational attainment?**

- High school
- Professional studies
- Collegial
- University (undergrad)
- University (Master)
- University (PhD)
- University (postdoc)

1. **What year was your program founded?**

**_____________________________________________________________________**

1. **Is it still running? If not, mention the year the program ended.**

- Yes
- No: Ending year: __________

1. **What is the largest scale at which your project is (was) operating?**

- Local
- Regional
- National
- International

1. **Location of data collection? More than one choice is possible.**

- North America: is it in a specific country or region? _______________________
- South America: is it in a specific country or region? _______________________
- Central America: is it in a specific country or region? ______________________
- Europe: is it in a specific country or region? ______________________
- Russia: is it in a specific region? ______________________
- Asia: is it in a specific country or region? ______________________
- Africa: is it in a specific country or region? ______________________
- Worldwide

1. **What is (was) the focal taxon for your project? More than one choice is possible.**

- Non-flying mammal
- Flying mammal
- Bird
- Reptile
- Amphibian
- Marine mammal
- Fish
- Insect

1. **What is (was) the type of data collected? More than one choice is possible.**

- Locations where the species has been observed
- Locations where the species reproduces
- Counts of individuals
- Counts of colonies or group of individuals
- Absence as well as presence of individuals
- Indirect presence (i.e. call, pellet, footprint, etc.)
- Other: _____________________________________________________

1. **What is (was) the main goal(s) of the project? More than one choice is possible.**

- Advancement of scientific knowledge (Distribution, ecology, population dynamics, etc.)
- Conservation
- Dissemination of results to the general public
- Public education and engagement

1. **Protocol and participant training.**
2. **Is (Was) a written protocol freely available?**

- Yes
- No

Comments: ____________________________________________________________________________________________________________________________________

1. **Did you use one or more rounds of pilot testing of the protocol before officially collecting data through this protocol.**

- Yes
- No

Comments: ____________________________________________________________________________________________________________________________________

1. **Is (Was) personal training/support freely available (i.e. data collection, organism identification, use of equipment)? More than one choice is possible.**

- In-person support (i.e. training or workshop)
- Hard copy support (i.e. pamphlets or guide)
- Electronic support (i.e. webpages or downloadable materials)
- None

Comments: ____________________________________________________________________________________________________________________________________

1. **How systematic are (were) the task procedures and data entry (e.g. for online data entry, do fields enforce type (e.g. counts are integers), and for categorical variables, users select from lists rather than entering free-form text)?**

- Systematic (only check boxes and/or items selected from lists except for comments)
- Semi-systematic (a mix of check boxes, lists, enforce type and free-form text)
- Non-systematic (only free-form text)

Comments: ____________________________________________________________________________________________________________________________________

1. **Does (Did) the project record relevant metadata? More than one choice is possible.**

- Environmental conditions (temperature, precipitation, time of day, etc.)
- Equipment or device settings (such as mobile device operating system version)
- Characteristics of the volunteers themselves (such as the level of education or training)
- None
- Other: ________________________________________

1. **Is (Was) there a collection feature allowing for data verifiability? More than one choice is possible.**

- Submission of photos
- Submission of audio
- Submission of specimens
- Expert present at the time of collection
- None
- Other: ______________________________________________________

1. **Is (Was) there data validation by a professional after the participant data entry?**

- Yes
- No

Comments: ____________________________________________________________________________________________________________________________________

1. **Is (was) collection effort standardized or accounted for in data analysis? I.e., were data standardized for inter-individual variation, effort, time, or distance?**

- All (all data collection methods were standardized)
- Some (only a portion of data collection methods were standardized)
- None
- Not applicable (no data analysis yet)

Comments: ____________________________________________________________________________________________________________________________________

1. **Database size.**
2. **What is (was) the total numbers of;**

**Sites? ___________**

**And/or**

**Counts? __________**

**And/or**

**Observations? __________**

**And/or**

**Other? Please, describe: __________**

1. **What is (was) the mean number of new entries per year of;**

**Sites? ___________**

**And/or**

**Counts? __________**

**And/or**

**Observations? __________**

**And/or**

**Other? Please, describe: _________**

1. **Was (Were) peer-reviewed publications produced using the project data?**

- None that I am aware of
- 1
- 2-5
- 6-10
- >10

**If the answer is yes, please, include the full title(s) and/or the link(s) of the paper(s) if possible.**

**________________________________________________________________________**

1. **Was (were) grey literature(s) (i.e. government reports, conference proceedings, conference abstracts, book chapters, theses, or magazine articles) produced using the project data?**

- None that I am aware of
- 1
- 2-5
- 6-10
- >10

**If the answer is yes, please, include the full title(s) and/or the link(s) of the paper(s) if possible.**

1. **Do (Did) conservation measures (e.g. conservation initiatives, management decisions, or policy actions) result from the project data?**

- None that I am aware of
- 1
- 2-5
- 6-10
- >10

**If the answer is yes, please, describe the measure(s).**

**________________________________________________________________________**

1. **What type of organizations runs (ran) the project? More than one choice is possible.**

- Governmental. Name the organization:____________________________
- Academic. Name the organization:________________________________
- Industrial. Name the organization:________________________________
- Non-profit Organization. Name the organization:____________________
- Citizen-driven organization. Name the organization:__________________

1. **What is (was) the disciplinary type? if your disciplinary type has changed over time, please checked your latest type and mention when and what was your previous type in the comments.**

-
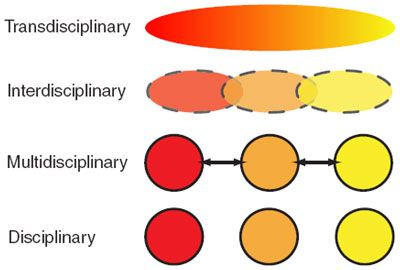
Intradisciplinary

Working within a single discipline (i.e. biology).

- Multidisciplinary

People from different disciplines working together, each drawing on their disciplinary knowledge in an additive manner (i.e. studying bats from the morphology and disease-carrying point of view).

- Interdisciplinary

Integration of knowledge and methods from different disciplines using a synthesis of approaches with stronger level of cooperation.

© Photo from nature.com

- Transdisciplinary

Creating a unity of intellectual frameworks beyond the disciplinary perspectives to form a new holistic approach.

Comments: ____________________________________________________________________________________________________________________________________

1. **What is (was) your governance structure? if your structure has changed over time, please checked your latest type and mention when and what was your previous structure in the comments.**

Definitions based on Conrad & Hilchey, 2010.

- Consultative/functional

Most often government led, community run; government recognizes problem and uses community-based monitoring group to monitor. Traditionally referred to as top-down.

- Collaborative/multi-party

Often governed by a board or group representing as many facets of the community as possible: private landowners, the general public, businesses, government, universities, etc. i.e. watershed.

- Transformative

Community led, run, and funded. Governed from the “bottom-up”. Also called community-based, grassroots, or advocacy groups. Community focuses on an issue with the hopes of initiating government action. Often focuses on specific local issues with no private sector or government support.

Comments: ____________________________________________________________________________________________________________________________________

1. **What is (was) the methodological approach of the project? If you check more than one choice because your project has many components, please explain it in the comments.**


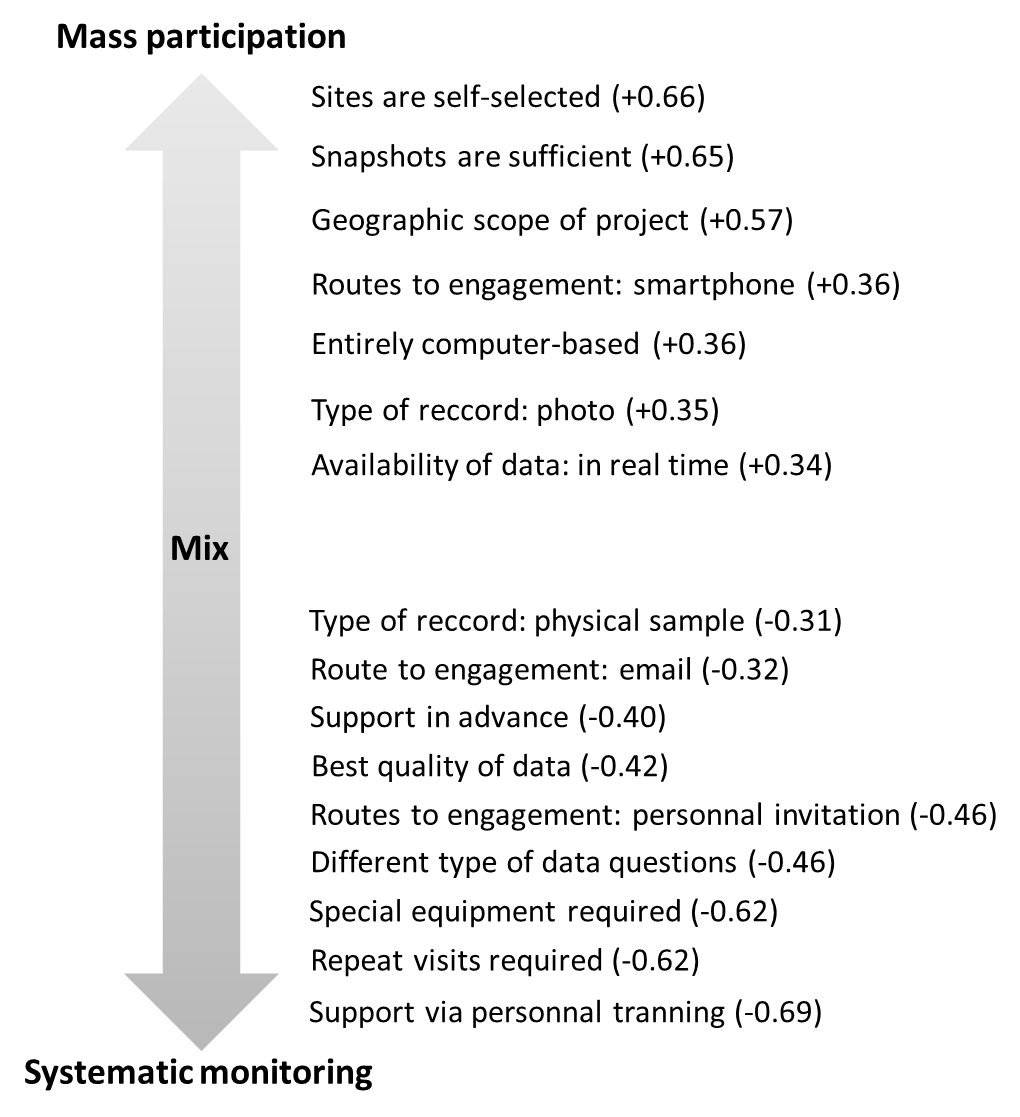
Figure based on Pocock & al. 2017 (table 1, axis 1).

- Mass participation

Tended to be projects in which anyone can get involved anywhere.

- Mixed
- Systematic

Tended to require participation at pre-defined sites that are visited repeatedly and to require particular equipment e.g. binoculars or tape measures.

Comments:

_______________________________

______________________________

______________________________

______________________________

1. **What is (was) the methodological complexity of the project? If you check more than one choice because your project has many components, please explain it in the comments.**

Gradient based on Pocock & al. 2017 (table 1, axis 2).

-
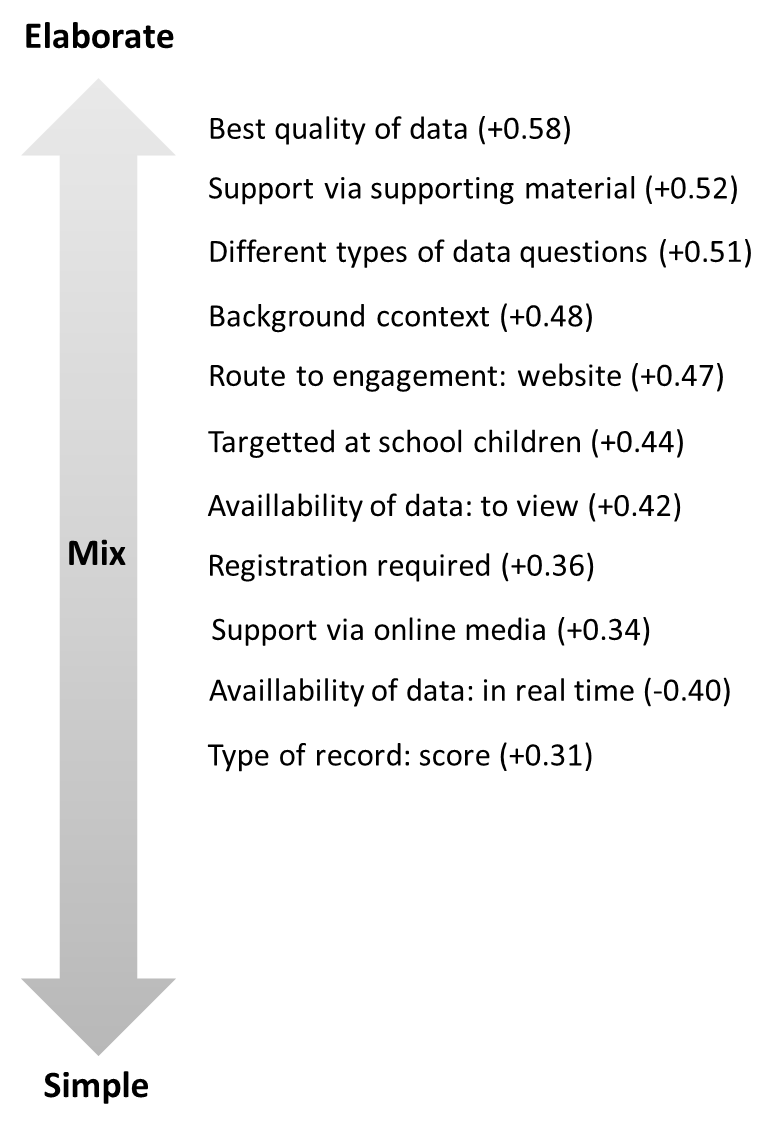
Elaborate

Tended to have complex protocols coupled with comprehensive supporting material and yield comparatively rich datasets.

- Mixed
- Simple

Tended to have little or no structured protocol, although they may require expertise such as species identification, and produced big datasets with simple structures.

Comments:

______________________________

______________________________

______________________________

______________________________

1. **If you win the pre-paid card price, can we mention your name and organization as winner in the participation thank you email?**

- **Yes**
- **No**
